# An integrative perspective on interorganizational multilevel healthcare networks: a systematic literature review

**DOI:** 10.1186/s12913-022-08314-6

**Published:** 2022-07-18

**Authors:** Galina van der Weert, Katarzyna Burzynska, Joris Knoben

**Affiliations:** 1 Radboud University Nijmegen; Institute for Management Research, Elinor Ostrom Building, Heyendaalseweg 141m, 6525 AJ Nijmegen, The Netherlands; 2 Tilburg University; Tilburg School of Economics and Management, Warandelaan 2, 5037 AB Tilburg, The Netherlands

**Keywords:** Network theory, Multilevel healthcare networks, Systematic literature review, Governance, Structure

## Abstract

**Background:**

Interorganizational networks in healthcare do not always attain their goals. Existing models outline the factors that could explain poor network performance: governance; structure; and the alignment of professional, organizational and network levels. However, these models are very generic and assume a functional approach. We investigate available empirical knowledge on how network structure and governance relate to each other and to network performance in a multilevel context, to get deeper insight, supported with empirics, of why networks (fail to) achieve their goals.

**Method:**

A systematic literature review based on a search of Web of Science, Business Source Complete and PubMed was executed in May 2021 and repeated in January 2022. Full papers were included if they were written in English and reported empirical data in a healthcare interorganizational setting. Included papers were coded for the topics of governance, structure, performance and multilevel networks. Papers from the scientific fields of management, administration and healthcare were compared. Document citation and bibliographic coupling networks were visualized using Vosviewer, and network measures were calculated with UCINET.

**Results:**

Overall, 184 papers were included in the review, most of which were from healthcare journals. Research in healthcare journals is primarily interested in the quality of care, while research in management and administration journals tend to focus on efficiency and financial aspects. Cross-citation is limited across different fields. Networks with a brokered form of governance are the most prevalent. Network performance is mostly measured at the community level. Only a few studies employed a multilevel perspective, and interaction effects were not usually measured between levels.

**Conclusions:**

Research on healthcare networks is fragmented across different scientific fields. The current review revealed a range of positive, negative and mixed effects and points to the need for more empirical research to identify the underlying reasons for these outcomes. Hardly any empirical research is available on the effects of different network structures and governance modes on healthcare network performance at different levels. We find a need for more empirical research to study healthcare networks at multiple levels while acknowledging hybrid governance models that may apply across different levels.

**Supplementary Information:**

The online version contains supplementary material available at 10.1186/s12913-022-08314-6.

## Introduction

An ageing population, medical and technological innovations and substantial knowledge about chemical, (bio) medical and technological fields have made the interplay between health conditions and treatment options increasingly complex [1, 2]. A direct consequence of this complexity is that professionals are increasingly becoming specialized while the provision of healthcare has become severely fragmented and characterized by many patient referrals and transfers between professionals and organizations [3–5] – with the risk of decreasing quality of care against increasing costs.

To optimize healthcare delivery and overcome obstacles related to fragmentation, healthcare organizations have been collaborating in many ways. This collaboration increasingly takes place in healthcare networks as groups of professionals and organizations work together, not only achieve their own goals better, cheaper and faster, but also to achieve a set of common goals [6, 7], such as improving the quality of care in settings where patients are surrounded by multiple healthcare providers. Collaboration is expected to yield positive results for the patient, the organization and society; a smoother patient journey characterized by fewer transfers or referrals; increased legitimacy, resource acquisition and economies of scale because equipment can be shared and used for a larger population; and, ultimately, cheaper healthcare provision according to the principles of value-based healthcare [8].

Theoretical models in the literature provide descriptions and conditions of organizational networks, such as the networks of professionals and organizations in healthcare. Provan and Milward suggested a model in which network structure and network context jointly affect network effectiveness [9]. According to this model, a combination of centralized integration and direct, non-fragmented external control, together with a stable system context and access to resources, would increase network effectiveness. More recently, a four-dimensional model of collaboration was proposed, by which interactions between governance, shared goals and vision, internalization and formalization are intended to enhance collaboration quality [10]. Furthermore, Provan and Kenis mention the importance of goal congruence, trust and governance [7]. They express that network governance should suit the network size, level of trust, amount of goal congruence and the need for network-level competencies within the network. Last, effective networks are characterized by their multilevel nature, because relationships are formed between professionals and organizations [11]. Members of networks should be selectively integrated, meaning that there should be a mix of close connections as well as ‘structural holes’ – parts of the network that are tied loosely together to maintain differentiation. This requires an appropriate form of coordination (governance): networks benefit from having a strong, central core while maintaining a flexible section of less connected members, that keeps the network open to new stakeholders and, hence, to new ideas [11].

All these theoretical models signify a properly performing network based on the set of contingency factors they describe. An appropriate form of governance is necessary to allocate resources, acquire legitimacy, arrange goal congruence within the network, address conflict, encourage actors, coordinate activities, prevent redundancy, constrain individual actors from hampering the process or stimulate members to look for new opportunities with partners. The form of governance is deemed appropriate when it fits the network’s size, level of goal consensus and the need for coordination within the network structure [7]. Network structure is reflected in the level of centralized integration and an optimal amount of formalization, and is often represented by network structural measures such as centrality, centralization and density [12]. A large, centralized network, where most of the partners are connected to just one or few other partners, requires a different governance approach than a small, decentralized network, where most partners are connected to each other [7, 13]. Larger, centralized networks encounter different problems than small, decentralized networks. Network members may bring different perspectives, norms and workflows with them, that need to be aligned: the greater the variation, the greater the need for coordination [7]. This is in line with Akmal & Gauld’s [14] description of Alliances Governance (AG), that ‘brings together elements of top-down vertical and horizontal cross-sector governance with the key difference that the aim is to bring together different actors in the health system. ( … ) The arrangement is contingent on commitment to building trust and sharing responsibility for system improvement.’. Specifically, members in an AG setting are expected to collaborate towards achieving a common goal with a focus on the whole system – as opposed to just their own organisation [15].

### Limitations of current theoretical models

Even though much work has been done on the topics of governance or structure in networks, the current knowledge is still limited. An often-overlooked factor is that the fit between governance and network structure may vary at different levels of an interorganizational network. Governance and structure on one level influence activities and outcomes on the other [5]. So far, research in this area has mainly focused on collaboration either at the patient, professional, organization or network level; if multiple levels are included, their connections are not investigated. This results in an incomplete picture of how networks function and how organizations collaborate. Owing to such low empirical support [16], we do not know how governance and structure affect each other at different levels of interorganizational networks.

A second limitation of these theoretical models is that they provide a functional view of network performance. The right constellation of contingency factors will have a perfect fit and ensure positive results. However, these perspectives are very generic and generally receive very little empirical support [17].

Networking does not resolve all existing problems in healthcare delivery; it affects the patient, the organization and society, and brings forth new problems that coincide with collaboration. Individual or organizational goals or tasks may conflict with network goals or even become redundant, and commitment to the network and its goals varies. Outcomes are affected by the interactions between and within the different levels [6, 18, 19]. People are used to working in hierarchies instead of across organizational boundaries and hierarchies, but healthcare networks are characterized by multiple managerial roles instead of having only one manager. Mechanisms such as power abuse, accountability and a lack of expertise and resources may also hamper collaboration [20]. Organizational cultures can clash, and professionals and organizational representatives may experience a loss of autonomy. Finally, networking implies increased coordination costs [11].

In summary, networks are increasingly used to solve problems in the healthcare sector. Even though theoretical models describe sets of contingency factors that can be considered to design a well-functioning network, many networks do not reach their goals sufficiently. Empirical insights need to be considered to develop a deeper understanding of when and why networks reach their goals or fail.

Relevant fields of literature on network research include healthcare, management sciences and public administration. Integrating the insights provided in these fields represents an important step toward explaining how and why networks reach – or fail to reach – their goals. Integrating these insights could also show organizations how to improve collaboration to achieve their goals.

So far, reviews have focused only on the role of governance in hospital systems and networks while ignoring its relationship with network structure or the multilevel nature of healthcare networks. These reviews conclude that knowledge on the topic is incomplete, fragmented and sometimes even contradictory [21].

Therefore, we carried out the present literature review to add to this knowledge by investigating the role of governance and structure by using the governance typologies of Provan and Kenis [7] and evaluating studies that have applied social network analysis to depict network structure. Finally, we provide an integrative perspective on the governance and structure of multilevel healthcare networks based on literature in the fields of management, public administration and healthcare. Specifically, we intend to answer the following question: ‘How do the governance and structure of healthcare networks relate to each other and to network performance in a multilevel context?’. By answering this question, we aim to inform science and practice of how governance and structure, and the relation between them, are important mechanics in predicting network performance. We also intend to find out, whether there is any overlap between the different scientific fields (healthcare, management or administration), indicated by cross-referencing or citations.

## Methods

We conducted a qualitative systematic literature review, added with a quantitative assessment of frequencies, for example when counting the number of papers applying a specific methodology or how often phenomena were discussed. In spring 2021 we searched the PubMed, Business Source Complete and Web of Science databases for peer-reviewed articles concerning governance and structure in multilevel healthcare networks. We searched for articles published until May 2021 using the following query:

**Table:** 

(interorganizational OR interorganisational OR inter-organizational OR inter-organisational) AND (health OR “health care” OR healthcare) AND (network*) AND (governance OR structure OR multilevel)	

Filters were set to provide results in English only. After removing duplicates, we obtained 392 articles. We repeated this search in January 2022, with the filter set to ‘publication year 2021’ to update the dataset. We added 17 papers (Web of Science=9; PubMed=4; Business Source Complete=4) during this last search. PRISMA guidelines [22] were followed in this systematic review where applicable (Additional file 2: Appendix 2).

### Inclusion criteria

**Fig. 1:**
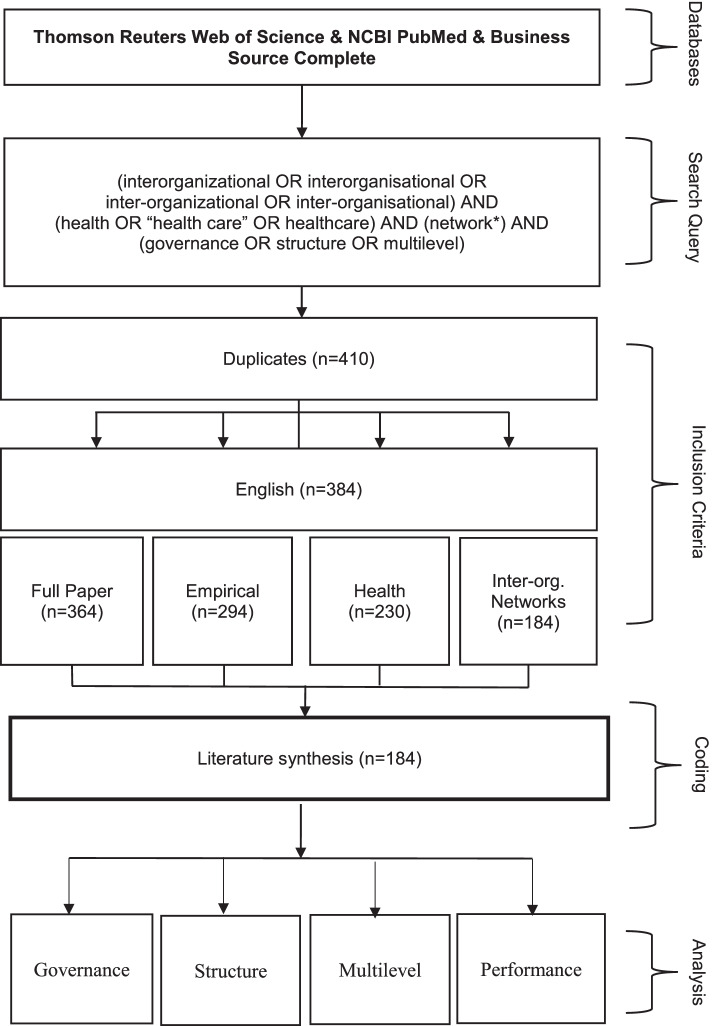
Inclusion criteria

All articles were subjected to a bibliographical analysis; the inclusion process is depicted in Fig. 1. We noted the author, title, year and field of publication (healthcare, administration, management, or other; derived from SSCI and NLM). Titles and abstracts were initially checked for eligibility for this review based on several criteria. First, we checked whether the article was available as a full text paper; study protocols and conference abstracts were excluded (*n=*28). Despite the language filter, 17 papers were written in a language other than English and were excluded. Next, we looked for empirical studies while excluding theoretical, conceptual or review papers (*n=*67).

Since the aim of this article is to analyse articles that concern collaboration between actors within the healthcare sector, studies that concerned other sectors were excluded, as were studies that did not have a healthcare network as the research subject (*n=*57) – for example studies that concerned telecommunications networks, information networks, biological or neurological (cell) networks and infrastructure networks. Our last inclusion criterion was that studies needed to investigate an interorganizational network. All studies related to collaboration on only a professional level or a patient level were excluded (*n=*45), as reviews on these levels are already available [19, 23, 24]. Alliances were included if they concerned three or more organizations, herewith adhering to our definition of an interorganizational network [6]– studies describing bilateral alliances were excluded. Papers that did not meet these criteria, were excluded from the analysis.

### Coding

Papers were coded using the theoretical background of interorganizational networks, including governance, structure and the level at which the research took place.

To code for governance, we used the commonly accepted typology of Provan and Kenis [7] to code the papers based on what form of governance was used in the networks. The first form of governance is shared governance, in which all participants have the same decision authority. The second form is a lead organization (LO), in which one organization takes the lead. The third form is a network administrative organization (NAO), in which a separate authority makes decisions and allocates resources. Each type has its own characteristics and is best for certain contexts [25].

Not all authors used the typology used by Provan and Kenis [7]. In these cases, we looked for a description of network governance within the article and tried to match these with the typology provided by Provan and Kenis [7]. While coding for mode of governance, we found that some papers described a combination of governance forms or hybrid forms. Thus, we created a new code (‘mix/hybrid’) to indicate such networks. For example, Wiktorowicz et al. [26] compared 10 networks, and not every network had the same mode of governance. Furthermore, Ciabuschi, Baraldi and Lindahl [27] described the complex governance characteristics of international multisectoral using a hybrid form of governance by which committees are responsible for decision making. This kind of organization resembles an NAO, but every committee is staffed with representatives from member organizations, which resembles a shared governance mode. In these cases, the code 'mix/hybrid’ was applied.

The measures used to depict the structure of the network (if any) were coded using terms derived from social network analysis, such as centralization, centrality and density measures [12]. Therefore, we first coded whether the paper applied social network analysis and then described the structure of the network.

For each paper, we coded at which level the study took place (professional, organizational, community or network) as in Provan and Milward [28]. The level of performance that was measured was coded (community, organization, network or combinations of these) as well as the specific performance outcome.

Finally, we coded papers to denote whether the research had a whole network perspective or studied a focal organization’s ego network [6]. We also examined what type of network the research concerned – either clinical care; a network arranged to organize healthcare for a specific disease, such as cancer care or a diabetes network; community care, public health, or other types, such as prevention, mental health, child and youth care.

All papers were initially coded by one researcher. Then, a random sample of 45 papers was also coded by two other authors. Due to the qualitative nature of the codes, specifically those related to network governance and performance, we approached intercoder reliability qualitatively, instead of calculating intercoder reliability ratings, which would not be very informative. Each author coded thirty papers such that there were fifteen papers that were coded by all authors. The consistency in the codes was discussed during a three-way meeting. Any inconsistencies were discussed, first by determining the reason for the inconsistency, then deciding how to solve it and, finally, choosing which code to apply. A code was changed only if all researchers agreed. The same strategy was then applied to the other 139 papers by the primary researcher.

For both network performance and network structure, the main differences in the provided codes were related to how strictly definitions were interpreted. For example, some papers did not provide a thorough description of how the network was governed. In such cases, two of the researchers tried to derive the governance mode from the description of the network, while the third would code it as ‘not applicable’ because governance was not a topic of interest of that paper.

We conducted a bibliometric analysis to investigate the extent of fragmentation across the research fields of interest, as was also done by Akmal, Greatbanks and Foote [29]. Specifically, Vosviewer [30] was used to visualize the document citation and bibliographic coupling network of the papers in the dataset. We did this by downloading the full record list with cited references from the core collection of Web of Science. Because we also used the PubMed and Business Source Complete databases, not all of the papers in these databases could be found in the Web of Science’s Core Collection. This is why 14 papers (7.61%) were not included in the network visualization. After importing the dataset in Vosviewer, we created a document citation network in which a connection exists between two nodes when they cite each other. The network file was imported in UCInet to calculate the centralization and density of the network [31].

Due to the great variety of the data, meta-analyses were impossible.

## Results

**Fig. 2:**
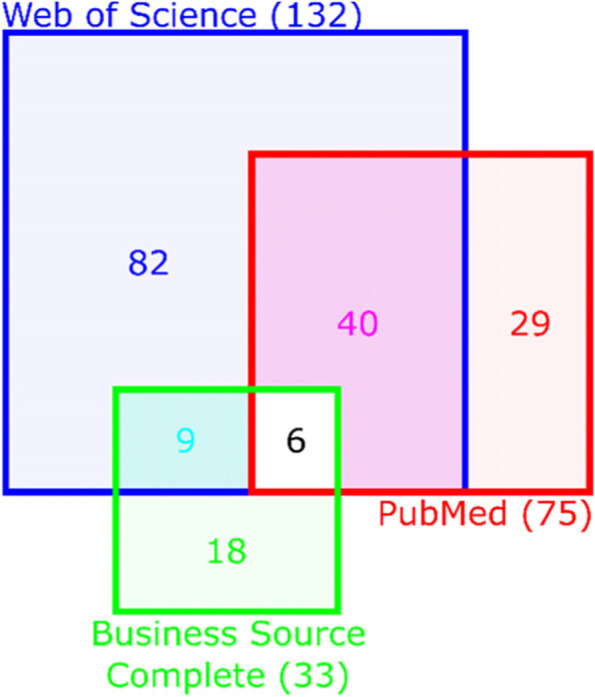
Division of search results from the Business Source Complete, PubMed and Web of Science databases

Ultimately, 184 papers were included in the data analysis (Fig. 2; see Additional file 1: Appendix 1 for an overview of all the papers that were included). We distinguished between papers found in PubMed, Web of Science, Business Source Complete or combinations of the three, to establish how much overlap there was among those databases. Figure 2 shows how many included papers were retrieved from each database. Six papers included in the data analysis were found in all three databases.

An overview of general results has been provided in Table 1. Healthcare journals are very well-represented in the dataset: 61% of the papers (*n=*109) were found in healthcare journals, whereas only 10% (*n=*19) were found in management journals, 8% (*n=*14) were found in administration journals and 21% (*N=*37) were found in other journals, such as those focusing on social networks (*n=*3) and evaluation and program planning (*n=*4). The majority of networks have a brokered form of governance (NAO or LO); NAO- and LO-governed networks are equally represented in healthcare and management journals. In administration journals, NAOs are more apparent than any other form of governance. This is expected, as governmental bodies very often either mandate, finance or coordinate the network. Due to a larger presence of the LOs compared to NAOs or shared governance networks in research in other journals than healthcare, management and administration, the LO form is more prominent in the total data-set (*n=*37).

**Table 1:** Overview of the number of papers per journal field, including the most important aspects (governance mode, multilevel, social network analysis applied, whole network perspective and type of network studied).

	Database		Governance form (n)		SNA?	Multilevel	Whole network	Network type		Topics	
Healthcare (*N=*111)	Web of Science (WoS)	42	NAO	16	51	27	100	Clinical care	14	Governance	46
	Business Source Complete (BuSCo)	5	LO	17				Community/local	24	Structure	64
	PubMed (PM)	21	Shared	8				Public health	12	Governance & structure	12
	WoS & BuSCo	0	Mix/hybrid	4				Prevention	8	Performance	65
	WoS & PM	38						Hospital	4		
	WoS, BuSCo & PM	5						Other	49		
Management (*N=*20)	WoS	8	NAO	3	5	5	16	Clinical care	2	Governance	13
	BuSCo	7	LO	4				Community/ local	5	Structure	10
	PM	0	Shared	1				Public health	1	Governance & structure	3
	WoS & BuSCo	5	Mix/hybrid	4				Prevention	0	Performance	13
	WoS & PM	0						Hospital	3		
	WoS, BuSCo & PM	0						Other	9		
Administration (*N=*15)	WoS	7	NAO	5	6	0	13	Clinical care	1	Governance	8
	BuSCo	4	LO	1				Community/ local	3	Structure	6
	PM	0	Shared	0				Public health	0	Governance & structure	4
	WoS & BuSCo	4	Mix/hybrid	1				Prevention	1	Performance	8
	WoS & PM	0						Hospital	1		
	WoS, BuSCo & PM	0						Other	9		
Other (*N=*38)	WoS	25	NAO	1	18	7	34	Clinical care	3	Governance	18
	BuSCo	2	LO	15				Community/local	7	Structure	24
	PM	8	Shared	1				Public health	2	Governance & Structure	11
	WoS & BuSCo	0	Mix/hybrid	1				Prevention	1	Performance	25
	WoS & PM	2						Hospital	4		
	WoS, BuSCo & PM	1						Other	21		
Total (*N=*184)	WoS	82	NAO	25	80	39	163	Clinical care	20	Governance	85
	BuSCo	18	LO	37				Community/ local	39	Structure	104
	PM	29	Shared	10				Public health	15	Governance & Structure	30
	WoS & BuSCo	9	Mix/hybrid	10				Prevention	10	Performance	111
	WoS & PM	40						Hospital	12		
	WoS, BuSCo & PM	6						Other	88		

In about 43.5% of the papers (*n=*78), social network analysis is used to depict the network structure. Papers in healthcare, administration and other journals have about the same proportions of studies that apply social network analysis (about 45%); meanwhile, network structure is not often measured with social network analysis in management journals (26.3%). Only 40 papers include multiple levels in their analysis. The proportion of multilevel analysis is comparable across all journal fields, except for administration, where only single-level analysis is applied. Most of the papers (*n=*158; 88%) apply a whole network perspective, which is comparable across all journal fields. Only in management is the proportion of research with a whole network perspective slightly lower (*N=*15; 79%).

In general, structure (*N=*102) and performance (*N=*106) are the most common themes addressed across all the journals. Most of the topics can be found in all journal fields, albeit in different proportions. In healthcare, most papers discuss the structure (58%) and performance (58%) of networks, while management journals discuss performance more (63%) while paying less attention to governance and structure (37% and 32%). In administration journals, governance and performance have garnered the most interest (50% of the papers discuss network governance, and 50% discuss performance). Both governance and structure are studied in 16% of the papers; however, examinations of governance and structure in multilevel networks are absent.

**Fig. 3:**
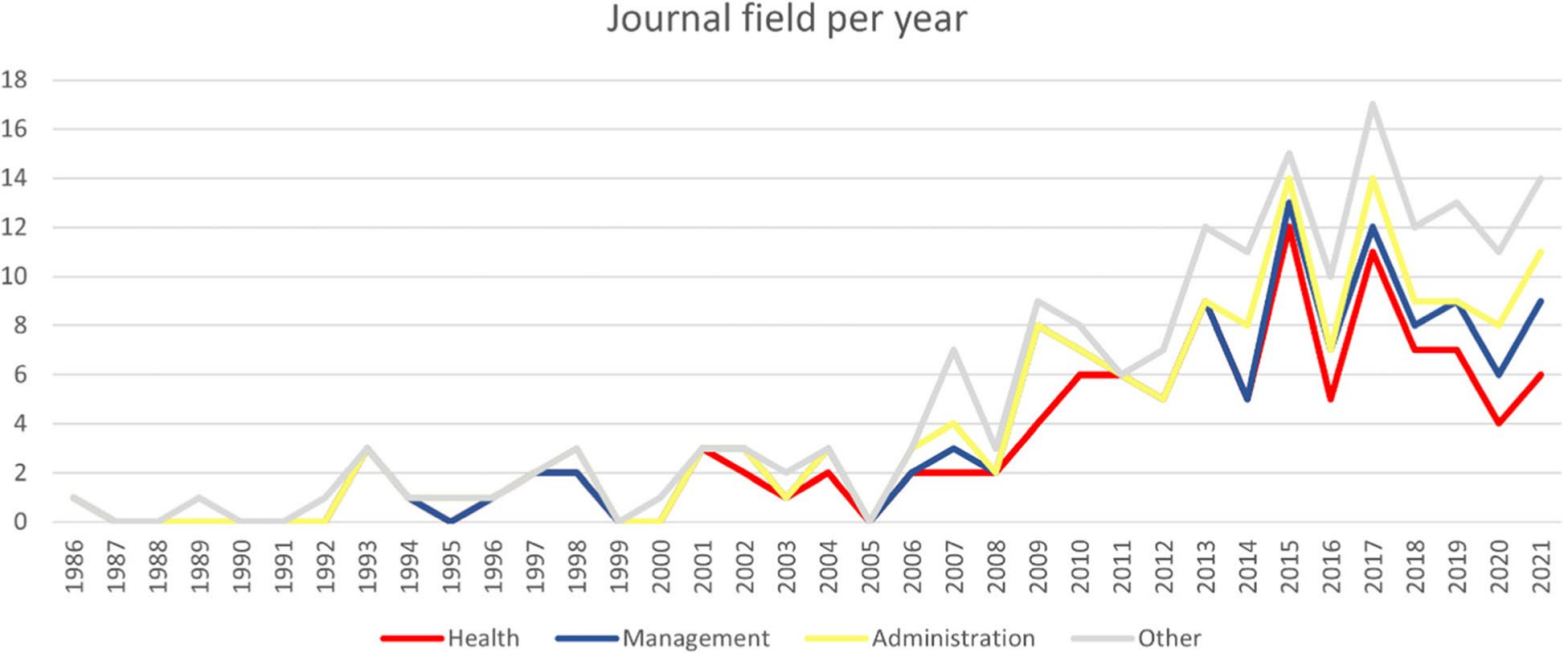
Number of publications per year and journal field

As we show in Fig. 3, the field was mainly covered by administration and ‘other’ journals. Provan & Milward’s paper [9] appeared in 1995 in an administration journal, but this theoretical basis and the interest in the topic was not picked up until later in the ‘90s and halfway through the ‘00s. Still, even though governance of healthcare networks is supposedly a topic of interest for a public administration as well as a healthcare audience, it has relatively little publications.

**Fig. 4:**
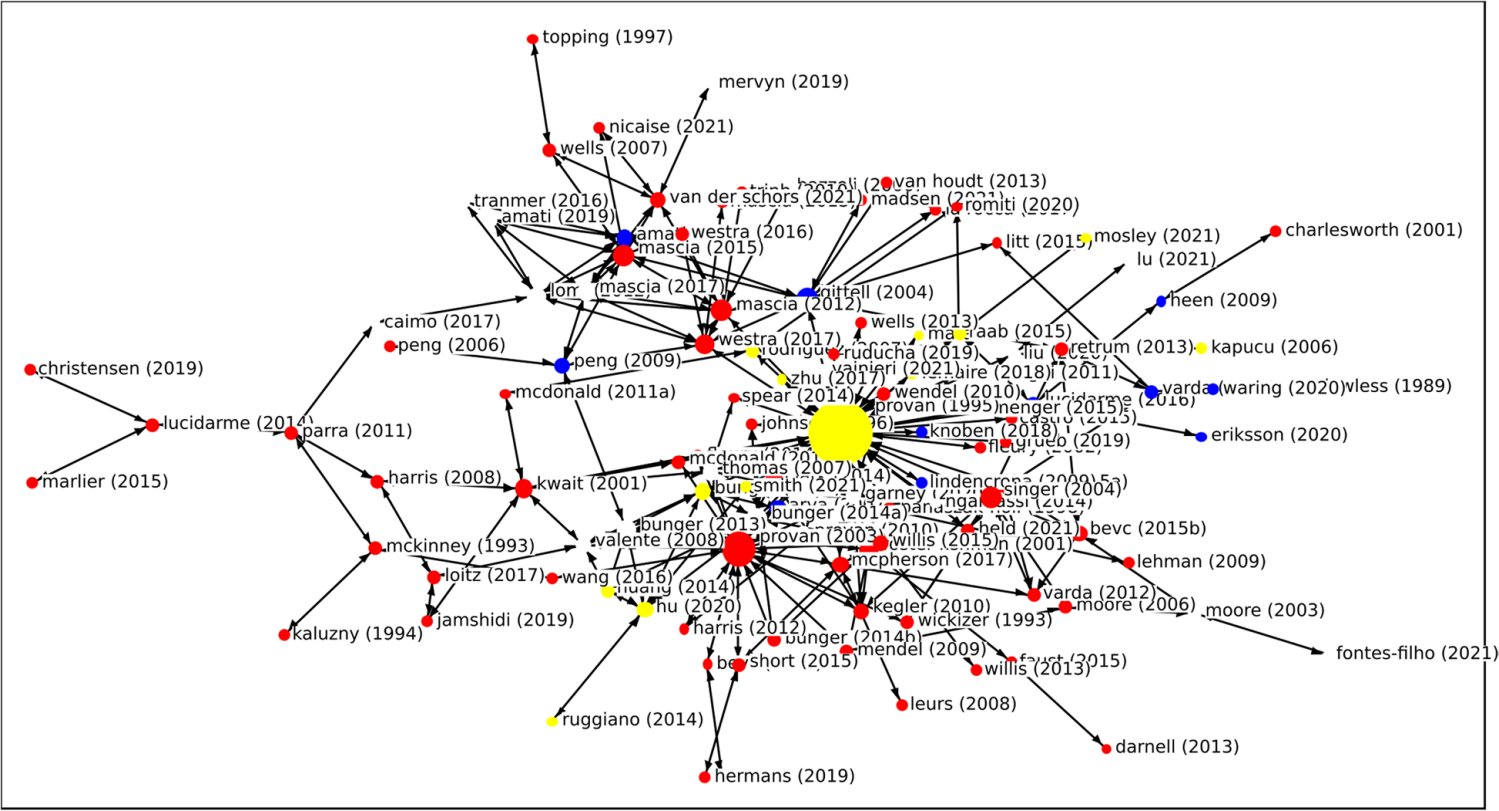
Citation network analysis. Journal field: red = healthcare; blue = management; yellow = administration; white = other

Knowledge about networks in healthcare is dispersed among the fields of healthcare, management and administration. This fragmentation is shown in Fig. 4, in the document citation network. The document citation network has a density of 0.033 – meaning that 3.3% of all possible ties are present, or that 3.3% of the documents in the dataset are cited by other documents in the dataset. Centralization is 0.358; meaning that 35.8% of ties runs through a single node. The paper with the highest indegree centrality, meaning the one that is cited the most by other papers in the dataset, is Provan & Milward [9]. The document citation network visualization as well as the network measures indicate that knowledge on the topics of governance and structure in multilevel networks is fragmented and dispersed, but there is a general knowledge base where management as well as healthcare research is based upon.

The bibliographic analysis further contained information on the number of papers published per year and most cited papers in each field; which journals publish most of the papers in each field; top publishing and top cited authors, and fields where those authors publish; top publishing and top citing organizations; and geographic affiliation. Because our analysis was already quite substantial, we decided to focus on the most important information, namely year of publication per field and top publishing and top cited authors, and the fields where these authors publish.

**Fig. 5:**
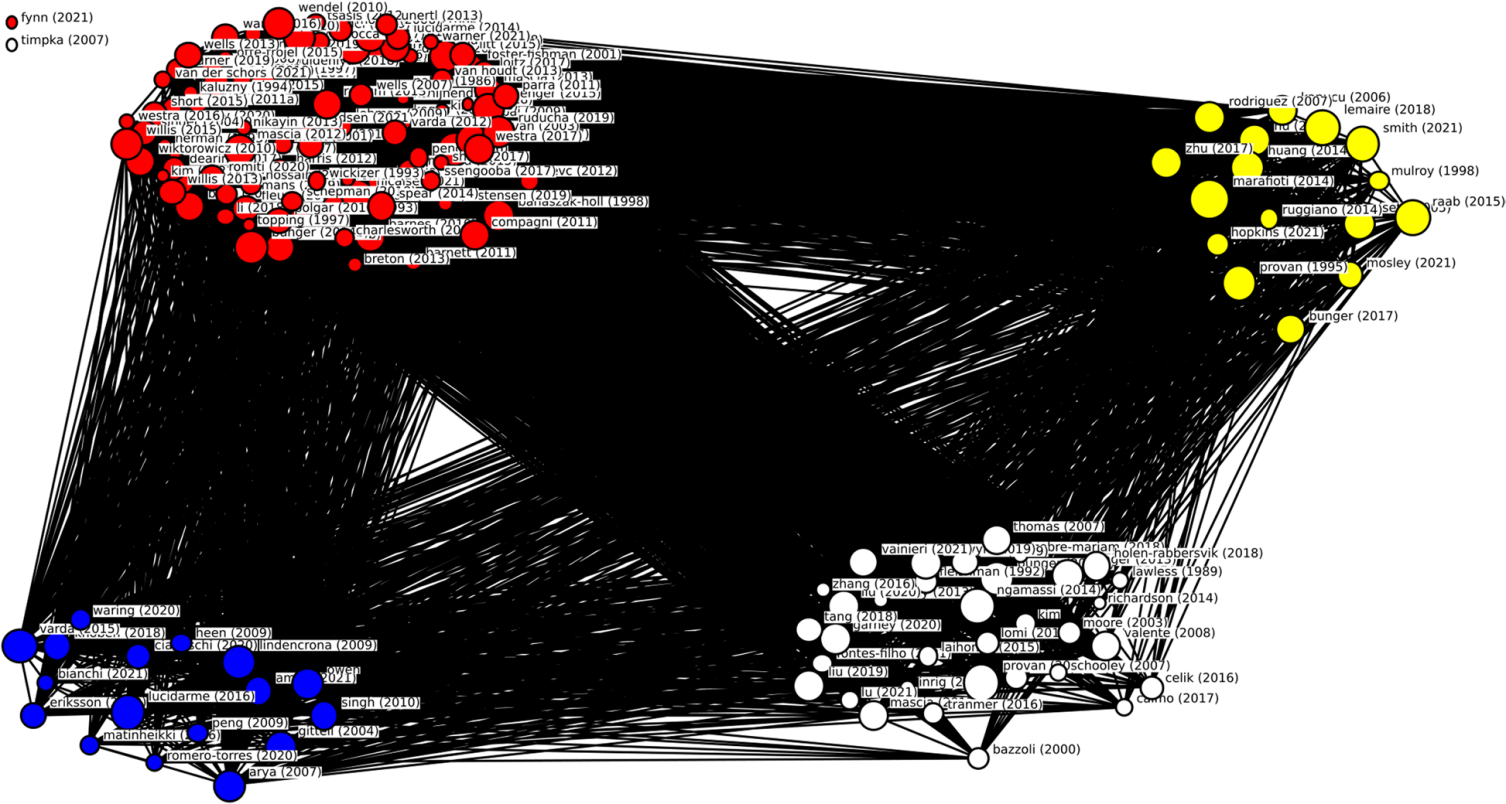
Visualization of the bibliographic coupling network, sorted per journal field

The document citation network was compared to a bibliographic coupling network (Fig. 5): a network that shows a link between two documents if they cite the same source. The bibliographic coupling network is much denser, roughly 35% of all papers in the dataset share the same references, than the document citation network, where only 3.3% of all papers cite each other. The articles in the dataset make use of a common knowledge base; i.e. they cite the same papers, but the low density of the document citation network shows that the articles rarely use each other’s knowledge. This could imply fragmentation in the field as authors build upon a small foundation of literature and use that to pursue different themes and phenomena.

### Performance

Network performance is often researched using a functional approach to address how the network can be organized to obtain optimal results. This means that all effects, impacts, outcomes or benefits are obtained because the individual partners started collaborating [32]. Network performance has also been referred to as network effectiveness, efficiency (usually financial performance) or network outcomes. For clarity, we use the term ‘network performance’ to refer to these various terms used to signify all types of network outcomes. Included articles show great variety in their use of performance measurements, perhaps unsurprisingly given the large variety in network types. Results are measured to obtain the (self-defined) network goal. To this end, one can define objective measurements or measure the perceived performance. Further, regarding ‘outcome’ measures, there is also a focus on process measures, the sustainability of collaborative workflows or problem-solving capabilities [33].

Network performance can be measured at three levels: the community level, the organizational level and the network level [9]. An overview of how performance was measured in our dataset is provided in Table 2. In general, the community level is the most prevalent in healthcare networks, specifically in healthcare journals, while management research predominantly focuses on performance at the network level. Performance measures at the community level are usually related to the reason for organizations’ initial collaboration (*n=*31) – for example, whether the number of babies born with low birthweight decreased due to collaboration [34]. Other, more generic, measures at the community level are quality of care (*n=*8), intervention implementation (*n=*9), decreasing readmission (*n=*2) and referrals, increasing awareness, patient safety, increasing access and increasing regional healthcare provision. At the network level, process outcomes are typically of interest, such as perceived quality of collaboration (*n=*15), which was the most apparent measure (for example, Nicaise et al. [35]).

**Table 2:** Performance levels per journal field.

	Community	Network	Organization	Combinations
Healthcare	33	24	3	5
Management	3	8	0	1
Administration	7	1	0	0
Other	12	10	1	2
Total	55	43	4	8

Next, communication and information sharing (*n=*8), administrative and financial coordination (including arrangements for funding) (*n=*7), building ties (*n=*2), increasing legitimacy (*n=*2), building trust (*n=*2) and learning and creating value were measured at the network level. Other factors that were found to affect network performance, are resource munificence [36], partner commitment [37] and variety of services or partner choice [38]. The continuity or availability of resources to support a network’s activities is not guaranteed, especially since most networks in healthcare depend on government funding or grants [36]. While heterogeneous networks have benefits, it poses coordination challenges and makes it difficult to achieve goal congruence among all stakeholders [39]. Furthermore, shared values and goal congruence are found to impact network performance positively, set expectations and make organizations feel that their efforts are needed and appreciated [40]. At the organizational level, an increase in financial performance or profits of the organization (*n=*3; for example, Bazzoli, Chan, Shortell and D’Aunno [41]) and patient satisfaction (*n=*1) were measured. Organizations with great resource availability invest in relationships and soon become more central actors in the network [42]. There is a strong ‘rich get richer’ phenomenon; actors without the opportunity or resources to invest in the network (either financially or in terms of human resources, time or equipment) become peripheral and benefit less than other actors. Additionally, in line with previous theories [7, 11, 28], system stability, organizational and network legitimacy and external contexts such as government involvement, policies and funding are vital for a network to reach its goals.

Three articles that studied local health networks observed performance at both the community and the network level. Such investigations are becoming increasingly important as Western healthcare searches for a balance between institutional health service provision and the self-reliance and resilience of patients and communities (for example, [43]). Other studies that combined performance measures at different levels did so to assess the quality of service provision and cost savings (*n=*4) and to compare the quality of service with the quality of collaboration (*n=*1 [44];). No studies compared performance at one level with performance at the other levels.

Network performance is negatively affected when goal congruence or commitment to network goals are difficult to achieve due to, for example, a lack of communication among partners. Administrative factors such as unclear hierarchy agreements also create performance barriers because accountability and responsibility issues impede collaboration. Too much formalization may simultaneously hamper integration and collaboration [45]. Network size can also be a barrier to network goals; the more complex a network structure becomes and the more active partners it contains, the more coordination is required [46]. When large networks perform well, it is typically related to sufficient variety in service providers; however, governing these networks is a complex endeavour [47]. Additionally, ineffective communication strategies across sectors appear to hamper a network’s ability to operate effectively [48].

Network age is another important factor in establishing performance [49]. In many cases, network performance is evaluated one or two years after the introduction of the network. This is not long enough for a network to mature and overcome problems related to early collaboration such as building trust, so the network’s actors are not yet ready to reach the network goals. The hurdles preventing effective collaboration must be considered first. Measurements of network performance after three years are much more informative. However, in the literature, networks are either assessed within one to two years after their emergence or after five or 10 years. These latter networks are quite often settled and have a history of reaching their goals – otherwise, they would not exist. None of the papers reviewed in this study researched networks that had failed and disassembled, which would have helped explain how and why networks fail.

### Structure

Centralized integration is supposed to be vital to increase network effectiveness [9]. The amount of centralized integration can be measured using social network analysis, together with network density and centrality of certain actors in the network. Overall, measures of centralization, centrality and density can provide a precise description of a network’s structure [12]. However, social network analysis is employed in only 43.5% of the papers reviewed in this study (*N=*78).

Network density refers to the number of links present in a network (expressed as a proportion of all possible links). Density increases over time in virtually all networks in the dataset. This may be because a greater variety of service providers joins the network over time, increasing the number of diagnosis and treatment options [50]. A higher density often means better performance because information spreads faster and actors find each other more easily [51]. Performance in a high-density network increases when a central coordinating body is in place, while dense networks with a lack of coordination soon experience redundancy [49].

Based on our dataset, we conclude that the density of a network depends on the type of network. Specifically, hospital networks are not especially complex [52] even though care provision is complex and hospital networks are horizontal and often small. Meanwhile, community care, elderly care and mental care networks are very complex systems encompassing great variety in terms of the partners involved and the services provided. Such networks are often characterized by mixed, nonhospital collaboration. This type of collaboration affects the number of links (and their strength) within the network. Density is also affected by geographical proximity; networks in which actors are geographically close to each other are denser than when actors are spread out across a region or country [53, 54].

Healthcare networks often struggle with the diversification vs integration dilemma [55]. Having a diverse array of partners is beneficial because it enhances treatment and diagnosis options. Integrating these services is important to prevent fragmentation, but such integration is complex given the great variety among partners. Thus, having a strong central governing body becomes more important as a network becomes more complex and denser, as centralized decision-making enhances network performance [41, 56]. As a network’s size increases, cliques (small, dense subgroups of actors) may form. Trust among actors within cliques is higher than within the entire network or between cliques [57]. A network that consists of many cliques also requires a strong governing body to maintain trust and, hence, effective collaboration between cliques.

It is generally assumed that centralization becomes more important as a network’s size increases, especially if such growth increases the diversity among partners [7]. However, the examined dataset shows that centralization alone does not always ensure high-quality collaboration. A possible explanation is that collaboration quality decreases not because of centralization but because of the variety of services represented, which makes it harder to achieve consensus [35]. Alternatively, the degree of formalization might not grow consistently with network size; thus, even if the network is centralized, it is challenging to achieve high-quality collaboration because a larger network does not necessarily require more centralization but more formalization [35, 58].

In a centralized network, the characteristics of the central organization are often that of a strong leader. The central organization often has access to many resources, and in many cases, the central organization is a governmental body, which is unsurprising because many networks are funded by the government [59]. This makes the central position complex; actors with a central position have to heavily invest resources in the network, but the resources also flow back to the organization, thereby increasing their ties and information flow and, in turn, making the central organization stronger [60]. Maintaining a central position is costly, which could negatively affect the central actor [42].

### Governance

Some form of governance is necessary for interorganizational networks, as governing collaboration increases network performance, either in terms of network goals or collaboration [44, 61]. Most networks are characterized by a brokered form of governance, with LOs (*n=*37; *n=*17 appeared in healthcare journals) being more prevalent than NAOs (*N=*25; *N=*16 in healthcare journals) or shared governance approach (*N=*10; *N=*8 in healthcare journals). The most common hybrid form of governance was a mix of NAOs and shared governance, especially in situations with very high substantive complexity, uncertainty and inequality [27].

In many cases, the network governance form fits with contingency factors [7]. An NAO is most effective when the network has moderate density, a moderate or large number of partners, moderately high goal consensus and a moderately high need for network-level competencies. In most NAO-governed networks, the characteristics were fitting. Specifically, they involved at least 10 agencies that provided multiple services (thus increasing the need for network-level competencies) and were usually centralized. In one case, though, only seven organizations were involved [62]. This network was built based on a federal grant to reduce child abuse in low-income areas. Still, this network was quite successful and was sustained for over five years. The grant purpose was achieved, agency expectations were met and the personal needs of participants were met. These goals were reached despite moderate investments from all stakeholders due to competent leadership and management. This example shows that strong central governance helps relatively small networks reach their goals.

An LO network should have a low density, be highly centralized, have a moderate or large number of partners, have moderately high goal consensus and a moderate need for network-level competencies [7]. An example can be found in a study in two hospital-led LO healthcare networks [63]. Network A has a strong, central leading organization, and its structure resembles that of a star: the leading organization is in the centre, surrounded by other network members. Compared to Network B, this network had a larger number of hospital beds, more contractual connections and a narrower scope; also, its structure was asymmetrical. Network B was led by the president of the central hospital but supported by an executive committee comprising organizational network members. Thus, Network B resembles a hybrid LO-shared governance mode. The connections and types of organizations within Network B are much more complicated than in Network A and contain more ownership connections (the president of the network owned many of the companies). Both networks competed and cooperated with each other; according to the conclusions, both networks and leading hospitals benefitted from improving cost-effectiveness, implementing innovations and maintaining public relationships [63].

Networks with a shared form of governance benefit from having high density, few partners, high levels of consensus and little need for network-level competencies [7]. Networks described as having shared governance mostly fit this description, with the exception of one local health network in Denmark [64]. This was a very loosely connected heterogeneous network that relied on the goodwill and voluntary investments of partners. The need for network-level competencies was high due to the great variation in structural differentiation, with external links either to governmental or non-governmental partners. Even though the network increased trust and communication among partners and achieved horizontal integration, it had relatively little success in achieving network goals such as organizing activities for the community it served. This may be because the network characteristics (high need for network-level competencies) were not aligned with the applied mode of governance (shared governance).

While it has been theorized that network governance should depend on the degree of interdependence in the network, in almost all cases in our dataset, a form of top-down centralized governance is used and found to be helpful in achieving goals. Previously discussed examples may have benefitted from adopting a brokered form of governance to deal with the complexities related to the variation of partners in the network [62, 64].

Which mode of governance is most effective may also depend on the type of network. Community-based care programs appear to be mostly governed by NAO forms of governance, while clinical care, emergency care and public health networks are governed by an LO. The benefits of a brokered form of governance – perhaps specifically an LO form – are that it builds trust and facilitates collaboration during stages that occur before network members have decided how to organize [65]. These benefits may be especially important for large, heterogeneous networks that have no history of collaboration or in situations with complex policy regulations – for example, public health networks that rely on a great variety of partners and deal with many different rules and regulations.

While having a strong central governing body increases overall network effectiveness, actually being the central actor can have benefits and costs for individual organizations. Being the central actor increases that actor’s influence in the network while providing better resources for their own organization [60]. But central organizations need to invest resources and capital. In many cases, network resources come from government funding, meaning governmental agencies often assume central or leading roles [66, 67]. According to our data, when governmental organizations took part in the network, they often had a central position and a governing role.

### Multilevel Networks

Healthcare networks are multilevel by nature. Collaboration happens at the clinical, professional, organizational and system levels, as well as at the community, organization and network levels [28, 68]. Furthermore, collaboration crosses functional and organizational boundaries and happens within and between these levels. Healthcare professionals collaborate with other professionals at the clinical level, but they also collaborate with patients and their families, and they provide input for policy at the organization and system levels. Similarly, policies made at the organization and system levels impact how professionals cooperate within and across organizations, as well as how they act towards patients and their families.

Therefore, it is important to employ a multilevel perspective when studying healthcare provision [69]. This notion has been highlighted in a study on child welfare networks, where information-sharing at the network level among agencies informed agreements at the level of the agency. Such agreements, in turn, increased coordination quality for individual children [70]. Some studies stress the characteristics of autonomy and occupational control in the medical profession or directly acknowledge conflicts between different levels [71, 72]. Nevertheless, they do not empirically study governance, structure or the differences between these factors at different levels of the network. Hence, potential misalignments are overlooked as possible explanations of disappointing network performance.

Most research on healthcare networks examines only a single level – either the network, organization, patient or professional level – while the multilevel aspect of interorganizational healthcare networks has not received much attention. In the entire dataset, only twenty articles employed a multilevel perspective. Furthermore, these studies are often limited to a ‘multiple’ level perspective, meaning that two or three levels are studied separately and are not linked to each other [36, 73]. Alternatively, the multilevel nature of the network is acknowledged, but the research question is addressed at only a single level. Importantly, no study on healthcare networks has combined network structure with a multilevel perspective despite the availability of network analytical tools – for example, the linked design approach [74]. However, because of the multilevel nature of networks, governance variations may exist at different levels, creating barriers to achieving network goals or affect the way changes or improvements are perceived at each level [9, 44].

Summarizing, assuming a multilevel perspective regarding the governance and structure in healthcare networks offers additional insight into why networks achieve their goals, or why they fail to do so.

## Discussion and implications

We started this investigation by following several theoretical models that describe the overall fit between network structure and governance in healthcare networks and how a good fit can ensure effective collaboration. In this section we integrate our findings, to indicate ways forward for the practice of networking in healthcare as well as for research in healthcare networks, regardless of the scientific field.

A comparison between research in healthcare, management and administration journals revealed that healthcare research is generally patient-oriented, while – unsurprisingly – research in management is organization-oriented. Bridging the gaps between these two streams of literature offers opportunities for enhanced engagement from both sides to improve research on healthcare networks, namely, by considering both the patient and organization or network perspective. For researchers in the management field, this means considering the patient perspective more. For instance, they should ask not only whether a network yields financial results or economies of scale but also whether the patient’s experience has improved.

Meanwhile, in the healthcare literature, we found that budgeting and financing are often considered to provide value-based healthcare; in other words, the goal is to provide the patient with the best results at the lowest possible cost [8]. However, the organizational perspective and network science perspective tend to be understudied. It still needs to be determined what network design yields the best result for the patient in terms of, for example, the quality of care or patient experiences. It also remains unknown whether a centrally steered network can improve the quality of care based on objective measurements and patient and professional experiences [9]. Another critical question that needs to be addressed is how individual healthcare organizations can benefit by collaborating in a network not only financially but also by increasing the quality of care and patient experiences.

Currently, the literature focuses mostly on structure and governance of healthcare networks. It is striking that only few papers pay attention to funding, and the power dynamics related to it. There are many reasons to study this in-depth, but it appears to be a tough topic to capture. Very little is known about the processes underlying the fit between structure and governance, such as the previous mentioned power dynamics but also psychological phenomena related to collaboration such as feeling of commitment and involvement [75].

### The interaction between structure and governance in multilevel healthcare networks: ways forward

We started by establishing that a form of governance is necessary in networks, to overcome the problems related to collaboration across professional and organizational boundaries. Different forms of network governance affect professionals and patients differently [9, 44]. Professionals are more satisfied when they are more autonomous, when there is less network steering and when they need to adhere to fewer protocols and procedures. Conversely, patients prefer strong steered networks with strict protocols. This situation indicates the need to incorporate different modes of governance at different levels of the network; a shared governance model can be integrated at the professional level within a larger, central and formalized governed network. Such a structure could enhance patient outcomes and professional satisfaction. Akmal and Gauld [14] add to this, stating that collaboration can only be successfully governed if the system surrounding the collaboration is congruent with what it expects from that collaboration; meaning that it should incentivize collaboration, there should be a general perspective on improving system level performance and there should be a focus on cross-sector and cross-service governance. In addition, the system surrounding the collaboration is important to explain the effectiveness of the network board or managing team; as Addicott [76] found, a collaboration that functions within a New Public Management System, finds that their boards only have marginal decision authority within the network, as opposed to clinical leaders and government authorities focused on output measures.

An example in which two modes of governance were combined successfully is provided by Ciabuschi, Baraldi [27], where an NAO form of governance at the network level was combined with shared governance at the level of committees. In this case, the centralized decision-making of the NAO accounted for rules, regulations, contracts and clear role definitions, while the professionals in the committees could contribute their expertise and specialization. In this specific study, the authors claimed that this combination was successful because of the complexity, uncertainty and urgency related to antibiotics resistance [27]. Based on the same arguments, this combination can be applied to the broader healthcare sector, which is also characterized by complexity, uncertainty and urgency.

Since we found ambiguity in structure’s and governance’s effects on network effectiveness at each level, we propose that, ideally, network performance should be measured at the patient, community, organization and network level together. Such a measurement requires network goals to be measurable. Additionally, before performance is measured, an agreement must be reached between network members and the institutions involved in the network, on how network performance will be measured – preferably after a longer period of time (3+ years after initiation) so that the network has time to mature [49].

Another important aspect of performance research in multilevel healthcare networks is the fact that alignment of performance across levels is missing. Although the performance of individual professionals or teams affects the performance of the whole network, this topic has not received much attention. Knowledge about how these two levels relate to each other and what mechanisms affect their mutual performance could help inform practice and research.

Social network analysis can be used to analyse and visualize network structures at both the organizational and professional levels, as well as to study the interdependencies between different network levels. Applying a network analysis at different levels of the network, allows evaluations of whether the professionals occupy a more or less comparable position in the network as the organization they represent, especially when they have a leading position [74]. Combining this knowledge with the way the network is governed at each level could provide insights into how networks can be adapted to perform better.

In short, it is important to consider the multilevel nature of networks, both in research and in practice. The patient and professional levels require a different approach than the organization and network levels concerning both governance and performance measurements. Insights into how organizational performances relate to community and network performance are especially important, as performance variations at different levels can highlight an uneven distribution in performance benefits received by single organizations, thus destabilizing the overall network.

In many networks, brokering or governance was executed by the most significant actor, who had access to many resources. This actor is often the largest organization in the network or a governmental body that has mandate or is considered the network’s leader. However, this organization may not be the best organization for the leadership role because it lacks the capacities or characteristics needed to govern the entire network. Different organizations may be more suitable to lead the network due to their identity or capacities. Also, if the largest actor (or the actor with access to the most resources) becomes the central actor in a network, it pushes other actors to the periphery, which induces a ‘rich get richer’ phenomenon. This situation may be detrimental to network effectiveness because the smaller, or specialized, organizations may also be needed to obtain the network’s goals. In addition, little is known about the organizational benefits of participating in networks. We assume that it is beneficial for organizations, especially those in LO-governed networks, to know whether the network benefits them in any way. For example, central organizations in networks saw their financial situations deteriorate [42].

### Limitations

When carrying out this review we encountered different forms of rhetoric, narratives and discourses in each scientific field. These differences were displayed by different uses of terminology, shown, for example, by the use of ‘integrated care’, ‘networked care’ and ‘collaborative care’ to describe any collaboration within and between professionals and organizations in healthcare delivery. This sometimes makes it difficult to compare findings, contexts or phenomena.

Not all modes of governance appeared equally in the dataset. The overrepresentation of brokered forms of governance (NAO or LO) could mean that this is the most effective way to govern healthcare networks, but it might also imply that shared governance networks are more difficult to research than NAOs and LOs, because they lack a specific leading organization that serves as a point of entry for researchers. NAOs and LOs often provide opportunities for larger datasets, because they often lead larger networks. There may be situations and contexts in which networks with a shared governance form are effective, but since they are not measured often, we do not know the specific characteristics that explain why they are effective. Furthermore, brokered networks with a NAO or LO form of governance are usually bounded networks in which membership is quite clear, making it easy to reach members through the brokering organization. A final factor explaining the overrepresentation of brokered forms of governance, is that much research is executed in the early stages of networks. Perhaps as networks evolve over time, a different form of governance becomes more apparent – or more hybrid forms are detectable [65]. Another explanation is that much of the research takes place in the Western world (the US and Europe), where a certain form of healthcare delivery is prevalent because it fits the politico-cultural context.

## Conclusions

In this systematic literature review, we have collected empirical data on the relationships among the governance, structure and performance of multilevel networks. We found that the fit between structure and governance – and its effects on network effectiveness – has not been studied thoroughly using empirical data. Relevant studies that we did find are fragmented and show inconclusive results. We conclude that, in most cases, having a centralized, brokered form of governance (an NAO or LO) yields better results for a network, regardless of the network structure. The benefits of centralization are the alignment of network activities, goal consensus, conflict resolution (and prevention) and resource allocation. However, centralized governance presents a risk of formalization, as over-regulation can impede individual partners’ discretion to execute tasks as they seem fit.

In summary, we call for a contextual, multilevel approach of network governance in healthcare networks that considers different governance needs and network structures at different levels in the network. Especially in times of crisis, such as the recent COVID-19 pandemic, there is a need to study different combinations of structure and governance in multilevel healthcare networks. This need is more pronounced now than ever due to the recent sudden increase in interorganizational, supra-regional collaboration with multiple governing layers involved in making decisions, coordinating activities and allocating resources. In addition, a way forward to develop our knowledge of why networks fail or succeed, is to go beyond the fit between governance and structure and look at power dynamics within relations and actors at multiple levels within the network. Given the findings that some organizations are more central than others, they may be in a situation or position where they can better use their power than other organizations, ultimately affecting the network’s effectiveness. Applying or adopting this new approach will also require new research designs, such as participatory action research or ethnography [77–79], to study the effects of governance and structure on the multiple levels within a network.

## Supplementary Information

**Additional file 1:** **Appendix 1.** Overview of all papers in the dataset.

**Additional file 2:** **Appendix 2.** PRISMA Guidelines.

## Data Availability

All data generated or analyzed during this study are included in this published article [and its supplementary information files].
