# Supplementary material for: An integrative perspective on interorganizational multilevel healthcare networks: a systematic literature review
Source: BMC Health Serv Res. 2022 Jul 18;22:923. doi: 10.1186/s12913-022-08314-6 (PMC9289349; doi:10.1186/s12913-022-08314-6)
Supplement: Supplementary file 1 — Additional file 1: Appendix 1. Overview of all papers in the dataset. [file 12913_2022_8314_MOESM1_ESM.docx]

Appendix 1. Overview of all papers in the dataset.

| Database | Journal | Journal field | year | DOI/PMID | Title | Author |
| --- | --- | --- | --- | --- | --- | --- |
| WoS | BMC HEALTH SERVICES RESEARCH | Health | 2011 | 10.1186/1472-6963-11-342 | Understanding innovators' experiences of barriers and facilitators in implementation and diffusion of healthcare service innovations: a qualitative study | Barnett, Julie; Vasileiou, Konstantina; Djemil, Fayika; Brooks, Laurence; Young, Terry |
| WoS; PM | JOURNAL OF HEALTH ORGANIZATION AND MANAGEMENT | Health | 2018 | 10.1108/JHOM-06-2017-0129 | Networks as systems: A case study of the World Health Organisation's Global Health Workforce Alliance | Best, Allan; Berland, Alex; Greenhalgh, Trisha; Bourgeault, Ivy L.; Saul, Jessie E.; Barker, Brittany |
| BuSCo | Public Money & Management | Management | 2021 | 10.1080/09540962.2019.1665361 | How does information system success come about in inter-organizational networks of public services? | Bianchi, Piervito; Trimigno, Mariangela |
| PM | BMC HEALTH SERVICES RESEARCH | Health | 2013 | 10.1186/1472-6963-13-262 | Reforming healthcare systems on a locally integrated basis: is there a potential for increasing collaborations in primary healthcare? | Breton M, Pineault R, Levesque JF, Roberge D, Da Silva RB, Prud'homme A. |
| PM | Supportive Care in Cancer | Health | 2015 | 10.1007/s00520-014-2451-5 | Identifying clinical and support service resources and network practices for cancer patients and survivors in southern Puerto Rico | Castro EM, JimÃ©nez JC, Quinn G, GarcÃ­a M, ColÃ³n Y, Ramos A, Brandon T, Simmons V, Gwede C, Vadaparampil S, Nazario CM. |
| BuSCo; WoS | ACADEMY OF MANAGEMENT PERSPECTIVES | Management | 2020 | 10.5465/amp.2019.0018 | Joining Forces to Prevent the Antibiotic Resistance Doomsday Scenario: The Rise of International Multisectoral Partnerships as a New Governance Model. | Ciabuschi, Francesco; Baraldi, Enrico; Lindahl, Olof |
| PM | Journal of Gerontology and Social Work | Health | 2020 | 10.1080/01634372.2020.1817828 | Interorganizational Context When Implementing Multisector Partnered Programs: A Qualitative Analysis of Veteran Directed Care | Clary AS, Perry KR, Edwards-Orr M, Miech EJ, VanHoutven C, Rudolph JL, Thomas KS, Sperber N. |
| PM | Health Services Management Research | Health | 2011 | 10.1258/hsmr.2010.010013 | The interplay between policy guidelines and local dynamics in shaping the scope of networks: the experience of the Italian Departments of Mental Health | Compagni, A; Gerzeli, S; Bergamaschi, M. |
| PM | BMJ Quality & Safety | Other | 2013 | 10.1136/bmjqs-2012-001166 | Competition in collaborative clothing: a qualitative case study of influences on collaborative quality improvement in the ICU. | Dainty KN, Scales DC, Sinuff T, Zwarenstein M. |
| WoS | HEALTH PROMOTION INTERNATIONAL | Health | 2017 | 10.1093/heapro/dat067 | How can the functioning and effectiveness of networks in the settings approach of health promotion be understood, achieved and researched? | Dietscher, Christina |
| BuSCo | Milbank Quarterly | Health | 2018 | 10.1111/1468-0009.12357 | The Generation of Integration: The Early Experience of Implementing Bundled Care in Ontario, Canada. | EMBULDENIYA, GAYATHRI; KIRST, MARITT; WALKER, KEVIN; WODCHIS, WALTER P. |
| WoS | PUBLIC MANAGEMENT REVIEW | Management | 2020 | 10.1080/14719037.2019.1604793 | Collaborative public management: coordinated value propositions among public service organizations | Eriksson, E; Andersson, T; Hellstrom, A; Gadolin, C; Lifvergren, S |
| WoS | INTERNATIONAL JOURNAL OF HEALTH PLANNING AND MANAGEMENT | Health | 2002 | 10.1002/hpm.684 | Regional planning implementation and its impact on integration of a mental health care network | Fleury, MJ; Mercier, C; Denis, JL |
| WoS | ANNALS OF PUBLIC AND COOPERATIVE ECONOMICS | Other | 2020 | 10.1111/apce. 12299 | Governance of interorganizational health emergency networks: Facing the Zika pandemic | Fontes, JR; Kaufmann, C; Fonseca, TM; Pimenta, R; de Souza, J; Novaes, E |
| BuSCo | Public Management Review | Management | 2009 | 10.1080/14719030802685263 | One Size Does Not Fit All | Heen, Hanne |
| PM | INTERNATIONAL JOURNAL OF INTEGRATED CARE | Health | 2019 | 10.5334/ijic.4186 | Integrated Palliative Care for Nursing Home Residents: Exploring the Challenges in the Collaboration between Nursing Homes, Home Care and Hospitals. | Hermans S, Sevenants A, Declercq A, Van Broeck N, Deliens L, Cohen J, Van Audenhove C. |
| WoS | PUBLIC ADMINISTRATION REVIEW | Administration | 2014 | 10.1111/puar. 12222 | Knowledge Sharing in a Third-Party-Governed Health and Human Services Network | Huang, Kun |
| PM | CHILD ABUSE & NEGLECT | Health | 2019 | 10.1016/j.chiabu.2018.10.013 | Professionals' preferences and experiences with inter-organizational consultation to assess suspicions of child abuse and neglect. | Konijnendijk AAJ, Boere-Boonekamp MM, Haasnoot ME, Need A. |
| WoS | AMERICAN REVIEW OF PUBLIC ADMINISTRATION | Administration | 2018 | 10.1177/0275074017700722 | Managing Collaborative Effort: How Simmelian Ties Advance Public Sector Networks | Lemaire, Robin H.; Provan, Keith G. |
| WoS | HEALTH POLICY | Health | 2018 | 10.1016/j.healthpol.2018.09.003 | Understanding inter-organizational trust among integrated care service provider networks: A perspective on organizational asymmetries | Li, Weizi; Islam, Aaquib; Johnson, Kevin; Lauchande, Priam; Shang, Xiaopu; Xu, Shen |
| WoS | PUBLIC MANAGEMENT REVIEW | Management | 2009 | 10.1080/14719030802685404 | Modes of Interaction and Performance of Human Service Networks A study of refugee resettlement support in Sweden | Lindencrona, Fredrik; Ekblad, Solvig; Axelsson, Runo |
| WoS | PUBLIC MANAGEMENT REVIEW | Management | 2016 | 10.1080/14719037.2015.1088567 | A COMPARATIVE STUDY OF HEALTH PROMOTION NETWORKS Configurations of determinants for network effectiveness | Lucidarme, Steffie; Cardon, Greet; Willem, Annick |
| WoS | INTERNATIONAL JOURNAL OF PUBLIC HEALTH | Health | 2014 | 10.1007/s00038-013-0527-5 | Critical success factors for physical activity promotion through community partnerships | Lucidarme, Steffie; Marlier, Mathieu; Cardon, Greet; De Bourdeaudhuij, Ilse; Willem, Annick |
| WoS | BMC PUBLIC HEALTH | Health | 2015 | 10.1186/s12889-015-2605-5 | Capacity building through cross-sector partnerships: a multiple case study of a sport program in disadvantaged communities in Belgium | Marlier, Mathieu; Lucidarme, Steffie; Cardon, Greet; De Bourdeaudhuij, Ilse; Babiak, Kathy; Willem, Annick |
| WoS | JOURNAL OF INTERPROFESSIONAL CARE | Health | 2011 | 10.3109/13561820.2011.566650 | Collaboration across private and public sector primary health care services: benefits, costs and policy implications | McDonald, Julie; Davies, Gawaine Powell; Jayasuriya, Rohan; Harris, Mark Fort |
| WoS | International Journal of Organizational Analysis | Other | 2019 | 10.1108/IJOA-05-2018-1415 | Challenges and Insights in inter-organizational collaborative healthcare networks | Mervyn, K; Amoo, N; Malby, R |
| WoS | JOURNAL OF PUBLIC ADMINISTRATION RESEARCH AND THEORY | Administration | 2021 | 10.1093/jopart/muaa044 | Decision-Making in Collaborative Governance Networks: Pathways to Input and Throughput Legitimacy | Mosley, Jennifer E.; Wong, Jade |
| WoS; PM | JOURNAL OF INTERPROFESSIONAL CARE | Health | 2020 | 10.1080/13561820.2019.1709425 | Key dimensions of collaboration quality in mental health care service networks | Nicaise, P; Grard, A; Leys, M; Van Audenhove, C; Lorant, V |
| WoS; PM | INTERNATIONAL JOURNAL OF MEDICAL INFORMATICS | Health | 2015 | 10.1016/j.ijmedinf.2015.07.001 | Developing patient portals in a fragmented healthcare system | Otte-Trojel, Terese; de Bont, Antoinette; Aspria, Marcello; Adams, Samantha; Rundall, Thomas G.; van de Klundert, Joris; de Mul, Marleen |
| BuSCo; WoS | ORGANIZATION STUDIES | Management | 2021 | 10.1177/01708406211031732 | Beyond the Crisis: Trust repair in an interorganizational network | Owen, Gareth; Currie, Graeme |
| BuSCo; WoS | Journal of Public Administration Research & Theory | Administration | 2015 | 10.1093/jopart/mut039 | Combining Structure, Governance, and Context: A Configurational Approach to Network Effectiveness. | Raab, Jörg; Mannak, Remco S.; Cambré, Bart |
| WoS | APPLIED CLINICAL INFORMATICS | Other | 2014 | 10.4338/ACI-2014-02-CR-0016 | A Case Report in Health Information Exchange for Inter-organizational Patient Transfers | Richardson, J. E.; Malhotra, S.; Kaushal, R. |
| WoS | BMC HEALTH SERVICES RESEARCH | Health | 2020 | 10.1186/s12913-020-05867-2 | Network governance forms in healthcare: empirical evidence from two Italian cancer networks | Romiti, Anna; Del Vecchio, Mario; Sartor, Gino |
| WoS; PM | BMC HEALTH SERVICES RESEARCH | Health | 2018 | 10.1186/s12913-018-3169-8 | Do project management and network governance contribute to inter-organisational collaboration in primary care? A mixed methods study | Schepman, Sanneke; Valentijn, Pim; Bruijnzeels, Marc; Maaijen, Marlies; de Bakker, Dinny; Batenburg, Ronald; de Bont, Antoinette |
| WoS; PM | HEALTH PROMOTION INTERNATIONAL | Health | 2015 | 10.1093/heapro/dau021 | Developing an inter-organizational community-based health network: an Australian investigation | Short, Alison; Phillips, Rebecca; Nugus, Peter; Dugdale, Paul; Greenfield, David |
| WoS | ADMINISTRATION & SOCIETY | Administration | 2020 | 10.1177/0095399720965556 | Strategic Orientation and Relationship Building Among Dyads in Complex Public Management Networks: Perspectives From State Asthma Coalitions | Smith, SL; Huang, K; Peng, SY |
| WoS | International journal of integrated care | Health | 2012 | 10-1-113786 / ijic2012-190 | Reframing the challenges to integrated care: a complex-adaptive systems perspective. | Tsasis, Peter; Evans, Jenna M; Owen, Susan |
| WoS | RESEARCH IN SOCIAL & ADMINISTRATIVE PHARMACY | Health | 2019 | 10.1016/j.sapharm.2018.09.015 | The role of network ties to support implementation of a community pharmacy enhanced services network | Turner, Kea; Weinberger, Morris; Renfro, Chelsea; Ferreri, Stefanie; Trygstad, Troy; Trogdon, Justin; Shea, Christopher M. |
| WoS | Sustainability | Other | 2021 | 10.3390/su13073873 | An Integrated Framework to Measure the Performance of Inter-Organizational Programme on Health Technology Assessment | Vainieri, Milena; Ferre, Francesca; Manetti, Stefania |
| WoS | PUBLIC MANAGEMENT REVIEW | Management | 2019 | 10.1080/14719037.2019.1588360 | The struggles for (and of) network management: an ethnographic study of non-dominant policy actors in the English healthcare system | Waring, Justin; Crompton, Amanda |
| WoS; PM | BMC HEALTH SERVICES RESEARCH | Health | 2021 | 10.1186/s12913-020-06031-6 | How do inner and outer settings affect implementation of a community-based innovation for older adults with a serious illness: a qualitative study | Warner, Grace; Kervin, Emily; Pesut, Barb; Urquhart, Robin; Duggleby, Wendy; Hill, Taylor |
| WoS; PM | INTERNATIONAL JOURNAL OF INTEGRATED CARE | Health | 2010 | 10.5334/ijic.525 | Mental health network governance: comparative analysis across Canadian regions | Wiktorowicz, Mary E.; Fleury, Marie-Josee; Adair, Carol E.; Lesage, Alain; Goldner, Elliot; Peters, Suzanne |
| BuSCo | American Journal of Public Health | Health | 2013 | 10.2105/AJPH.2013.301249 | Networks to Strengthen Health Systems for Chronic Disease Prevention. | Willis, Cameron D.; Riley, Barbara L.; Herbert, Carol P.;Best, Allan |
| WoS | ELECTRONIC JOURNAL OF INFORMATION SYSTEMS IN DEVELOPING COUNTRIES | Other | 2018 | 10.1002/isd2. 12045 | Governance lessons from an interorganizational health information system implementation in Ethiopia | Gebre-Mariam, Mikael |
| WoS | HEALTH EDUCATION RESEARCH | Health | 1993 | 10.1093/her/8.3. 331 | THE EVOLUTION, OPERATION AND FUTURE OF MINNESOTA SAFPLAN - A COALITION FOR FAMILY-PLANNING | HERMAN, KA; WOLFSON, M; FORSTER, JL |
| WoS | BMC MEDICAL INFORMATICS AND DECISION MAKING | Other | 2018 | 10.1186/s12911-018-0701-z | Barriers to exchanging healthcare information in inter-municipal healthcare services: a qualitative case study | Holen-Rabbersvik, Elisabeth; Thygesen, Elin; Eikebrokk, Tom Roar; Fensli, Rune Werner; Slettebo, Ashild |
| BuSCo | Human Service Organizations: Management, Leadership & Governance | Administration | 2020 | 10.1080/23303131.2019.1696908 | Professional Friendship, Resource Competition, and Collaboration in a Homeless Service Delivery Network. | Hu, Qian; Huang, Kun; Chen, Bin |
| PM | ASIA PACIFIC JOURNAL OF PUBLIC HEALTH | Health | 2018 | 10.1177/1010539518762847 | Dynamics of Interorganizational Public Health Emergency Management Networks: Following the 2015 MERS Response in South Korea | Kim K, Jung K. |
| PM | Risk Analysis | Other | 2021 | 10.1111/risa.13751 | Effectiveness of Emergent Ad Hoc Coordination Groups in Public Health Emergencies | Kim Y, Lee K, Oh SS, Park H. |
| PM | DISASTER MEDICINE AND PUBLIC HEALTH PREPAREDNESS | Health | 2021 | 10.1017/dmp.2020.32 | Interorganizational Coordination and Collaboration During the 2015 MERS-CoV Response in South Korea | Kim Y, Oh SS, Ku M, Byeon J. |
| WoS | INTERNATIONAL JOURNAL OF ENVIRONMENTAL RESEARCH AND PUBLIC HEALTH | Health | 2017 | 10.3390/ijerph14091064 | Public Health Network Structure and Collaboration Effectiveness during the 2015 MERS Outbreak in South Korea: An Institutional Collective Action Framework | Kim, KyungWoo; Andrew, Simon A.; Jung, Kyujin |
| WoS; PM | NATURAL HAZARDS | Other | 2020 | 10.1007/s11069-020-04379-w | Building the COVID-19 Collaborative Emergency Network: a case study of COVID-19 outbreak in Hubei Province, China | Liu, J; Hao, JY; Shi, ZW; Bao, HXH |
| PM | NATURAL HAZARDS | Other | 2021 | 10.1007/s11069-021-04908-1 | Dynamic analysis of emergency inter-organizational communication network under public health emergency: a case study of COVID-19 in Hubei Province of China | Lu, Yunmeng; Liu, Tiezhong; Wang, Tiantan |
| WoS | INTERNATIONAL JOURNAL OF PROJECT MANAGEMENT | Management | 2016 | 10.1016/j.ijproman.2016.06.003 | Managing inter-organizational networks for value creation in the front-end of projects | Matinheikki, Juri; Artto, Karlos; Peltokorpi, Antti; Rajala, Risto |
| WoS | INTERNATIONAL JOURNAL OF MEDICAL INFORMATICS | Health | 2013 | 10.1016/j.ijmedinf.2013.06.013 | Collective action for a common service platform for independent living services | Nikayin, Fatemeh; De Reuver, Mark; Itala, Timo |
| PM | JOURNAL OF HEALTH ORGANIZATION AND MANAGEMENT | Health | 2017 | 10.1108/JHOM-06-2016-0123 | The institutional logic of integrated care: an ethnography of patient transitions | Shaw JA, Kontos P, Martin W, Victor C. |
| WoS | GLOBALIZATION AND HEALTH | Health | 2017 | 10.1186/s12992-017-0251-7 | Sub-national assessment of aid effectiveness: A case study of post-conflict districts in Uganda | Ssengooba, Freddie; Namakula, Justine; Kawooya, Vincent; Fustukian, Suzanne |
| WoS | COMPLEXITY | Other | 2018 | 10.1155/2018/8935872 | Examining the Intergovernmental and Interorganizational Network of Responding to Major Accidents for Improving the Emergency Management System in China | Tang, Pan; Chen, Haojia; Shao, Shiqi |
| WoS | Psychiatric Quarterly | Health | 2013 | 10.1007/s11126-012-9245-z | How a Stressed Local Public System Copes With People in Psychiatric Crisis | Wells, Rebecca; Holdsworth, E.; Morrissey, J.; Hall, M.; Hassmiller, K.; Blouin, R. |
| WoS | SAFETY SCIENCE | Other | 2016 | 10.1016/j.ssci.2015.11.012 | The emergence of an adaptive response network: The April 20, 2013 Lushan, China Earthquake | Zhang, Haibo; Zhang, Xiaosu; Comfort, Louise; Chen, Mengyao |
| PM | Journal of public health management practice | Health | 2012 | 10.1097/PHH.0b013e31825fbaf9 | Assessing the roles of brokerage: an evaluation of a hospital-based Public Health epidemiologist program in North Carolina | Bevc CA, Markiewicz ML, Hegle J, Horney JA, MacDonald PD. |
| BuSCo; WoS; PM | American Journal of Public Health | Health | 2015 | 10.2105/AJPH.2014.302256 | New Perspectives on the "Silo Effect": Initial Comparisons of Network Structures Across Public Health Collaboratives. | Bevc, Christine A.; Retrum, Jessica H.; Varda, Danielle M. |
| WoS; PM | INTERNATIONAL JOURNAL OF ENVIRONMENTAL RESEARCH AND PUBLIC HEALTH | Health | 2015 | 10.3390/ijerph121012412 | Patterns in PARTNERing across Public Health Collaboratives | Bevc, Christine A.; Retrum, Jessica H.; Varda, Danielle M. |
| WoS; PM | INTERNATIONAL JOURNAL OF HEALTH PLANNING AND MANAGEMENT | Health | 2019 | 10.1002/hpm.2620 | Health in All local Policies: Lessons learned on intersectoral collaboration in a community-based health promotion network in Denmark | Christensen, Julie Hellesoe; Bloch, Paul; Moller, Signe Rysbjerg; Sogaard, Cecilie Pruesse; Klinker, Charlotte Demant; Aagaard-Hansen, Jens; Bentsen, Peter |
| PM | AMERICAN JOURNAL OF COMMUNITY PSYCHOLOGY | Health | 2013 | 10.1007/s10464-012-9559-x | Testing effects of community collaboration on rates of low infant birthweight at the county level | Darnell AJ, Barile JP, Weaver SR, Harper CR, Kuperminc GP, Emshoff JG. |
| WoS; PM | CADERNOS DE SAUDE PUBLICA | Health | 2017 | 10.1590/0102-311X00063516 | Strategic factors for the sustainability of a health intervention at municipal level of Brazil | de Araujo Oliveira, Sydia Rosana; Medina, Maria Guadalupe; Figueiro, Ana Claudia; Potvin, Louise |
| WoS; PM | IMPLEMENTATION SCIENCE | Health | 2017 | 10.1186/s13012-017-0542-7 | Pathways for best practice diffusion: the structure of informal relationships in Canada's long-term care sector | Dearing, James W.; Beacom, Amanda M.; Chamberlain, Stephanie A.; Meng, Jingbo; Berta, Whitney B.; Keefe, Janice M.; Squires, Janet E.; Doupe, Malcolm B.; Taylor, Deanne; Reid, Robert Colin; Cook, Heather; Cummings, Greta G.; Baumbusch, Jennifer L.; Knopp-Sihota, Jennifer; Norton, Peter G.; Estabrooks, Carole A. |
| WoS | PSYCHOSOCIAL INTERVENTION-INTERVENCION PSICOSOCIAL | Health | 2015 | 10.1016/j.psi.2015.09.002 | Exploring relationships among organizational capacity, collaboration, and network change | Faust, Victoria; Christens, Brian D.; Sparks, Shannon M. A.; Hilgendorf, Amy E. |
| BuSCo; WoS; PM | EVALUATION AND PROGRAM PLANNING | Other | 2020 | 10.1016/j.evalprogplan.2019.101771 | Interorganizational network findings from a nationwide cardiovascular disease prevention initiative | Garney, WR; Patterson, MS; Garcia, K; Muraleetharan, D; McLeroy, K |
| WoS | Journal of prevention & intervention in the community | Health | 2011 | 10.1080/10852352.2011.530168 | The urban context: a place to eliminate health disparities and build organizational capacity. | Gilbert, Keon L; Quinn, Sandra Crouse; Ford, Angela F; Thomas, Stephen B |
| WoS; PM | SOCIAL SCIENCE & MEDICINE | Health | 2008 | 10.1016/j.socscimed.2008.07.013 | Seeing the forest and the trees: Using network analysis to develop an organizational blueprint of state tobacco control systems | Harris, Jenine K.; Luke, Douglas A.; Burke, Ryan C.; Mueller, Nancy B. |
| WoS | IMPLEMENTATION SCIENCE | Health | 2012 | 10.1186/1748-5908-7-69 | Drawbacks and benefits associated with inter-organizational collaboration along the discovery-development-delivery continuum: a cancer research network case study | Harris, Jenine K.; Provan, Keith G.; Johnson, Kimberly J.; Leischow, Scott J. |
| WoS; PM | HEALTH PROMOTION JOURNAL OF AUSTRALIA | Health | 2021 | 10.1002/hpja.374 | Core and peripheral organisations in prevention: Insights from social network analysis | Held, F; Hawe, P; Roberts, N; Conte, K; Riley, T |
| WoS | PLOS ONE | Other | 2019 | 10.1371/journal.pone.0219786 | Using social network analysis to plan, promote and monitor intersectoral collaboration for health in rural India | Hoe, Connie; Adhikari, Binita; Glandon, Douglas; Das, Arindam; Kaur, Navpreet; Gupta, Shivam |
| BuSCo; WoS | International Journal of Public Administration | Administration | 2021 | 10.1080/01900692.2019.1669177 | Collaborative Networks in Chronic Disease Prevention: What Factors Inhibit Partnering for Funding? | Hopkins, Liza; Chamberlain, Daniel; Held, Fabian; Riley, Therese; Wang, Jean Zhou Jing; Conte, Kathleen |
| WoS; PM | DISASTER MEDICINE AND PUBLIC HEALTH PREPAREDNESS | Health | 2015 | 10.1017/dmp.2014.88 | Networks of Preparedness and Response During Australian H1N1 Outbreak | Hossain, Liaquat; Bdeir, Fadl; Crawford, John W.; Wigand, Rolf T. |
| WoS | EVALUATION AND PROGRAM PLANNING | Other | 2017 | 10.1016/j.evalprogplan.2016.11.006 | Assessing local capacity to expand rural breast cancer screening and patient navigation: An iterative mixed-method tool | Inrig, Stephen J.; Higashi, Robin T.; Tiro, Jasmin A.; Argenbright, Keith E.; Craddock, Simon J. |
| WoS; PM | MALARIA JOURNAL | Health | 2019 | 10.1186/s12936-018-2635-4 | A social network analysis on immigrants and refugees access to services in the malaria elimination context | Jamshidi, Ensiyeh; Ardebili, Hassan Eftekhar; Yousefi-Nooraie, Reza; Raeisi, Ahmad; Ardakani, Hossein Malekafzali; Sadeghi, Roya; Hanafi-Bojd, Ahmad Ali; Majdzadeh, Reza |
| WoS; PM | HEALTH EDUCATION RESEARCH | Health | 2010 | 10.1093/her/cyq022 | Using network analysis to assess the evolution of organizational collaboration in response to a major environmental health threat | Kegler, Michelle C.; Rigler, Jessica; Ravani, Maya K. |
| BuSCo; WoS | Organization Science | Management | 2018 | 10.1287/orsc.2017.1180 | What Do They Know? The Antecedents of Information Accuracy Differentials in Interorganizational Networks. | Knoben, Joris; Oerlemans, Leon A. G.; Krijkamp, Annefleur R.; Provan, Keith G. |
| BuSCo; WoS; PM | American Journal of Public Health | Health | 2015 | 10.2105/AJPH.2015.302828 | How to Identify Success Among Networks That Promote Active Living. | Litt, Jill; Varda, Danielle; Reed, Hannah; Retrum, Jessica; Tabak, Rachel; Gustat, Jeanette; O'Hara Tompkins, Nancy |
| WoS | JOURNAL OF APPLIED COMMUNICATION RESEARCH | Other | 2019 | 10.1080/00909882.2019.1620958 | Promoting collaboration: the role of relational multiplexity in an interorganizational health justice network | Liu, Wenlin; Beacom, Amanda M.; Frank, Lauren B.; Nomachi, Jonathan; Vasquez, Sonya; Galloway-Gilliam, Lark |
| WoS; PM | BMC PUBLIC HEALTH | Health | 2017 | 10.1186/s12889-017-4661-5 | Network analysis of inter-organizational relationships and policy use among active living organizations in Alberta, Canada | Loitz, Christina C.; Stearns, Jodie A.; Fraser, Shawn N.; Storey, Kate; Spence, John C. |
| WoS | BMC HEALTH SERVICES RESEARCH | Health | 2017 | 10.1186/s12913-017-2018-5 | A catalyst for system change: a case study of child health network formation, evolution and sustainability in Canada | McPherson, Charmaine; Ploeg, Jenny; Edwards, Nancy; Ciliska, Donna; Sword, Wendy |
| WoS; PM | HEALTH SERVICES RESEARCH | Health | 2009 | 10.1111/j.1475-6773.2008.00932.x | The Growth of Partnerships to Support Patient Safety Practice Adoption | Mendel, Peter; Damberg, Cheryl L.; Sorbero, Melony E. S.; Varda, Danielle M.; Farley, Donna O. |
| PM | Disasters | Other | 2003 | 10.1111/j.0361-3666.2003.00235.x | International NGOs and the role of network centrality in humanitarian aid operations: a case study of coordination during the 2000 Mozambique floods. | Moore S, Eng E, Daniel M. |
| WoS; PM | BMC HEALTH SERVICES RESEARCH | Health | 2006 | 10.1186/1472-6963-6-141 | The influence of partnership centrality on organizational perceptions of support: a case study of the AHLN structure | Moore, Spencer; Smith, Cynthia; Simpson, Tammy; Minke, Sharlene Wolbeck |
| WoS; PM | SOCIAL SCIENCE & MEDICINE | Health | 2011 | 10.1016/j.socscimed.2011.08.020 | How does network structure affect partnerships for promoting physical activity? Evidence from Brazil and Colombia | Parra, Diana C.; Dauti, Marsela; Harris, Jenine K.; Reyes, Lissette; Malta, Deborah C.; Brownson, Ross C.; Quintero, Mario A.; Pratt, Michael |
| PM | Journal of Substance Abuse Treatment | Health | 2010 | 10.1016/j.jsat.2009.12.008 | A longitudinal study of organizational formation, innovation adoption, and dissemination activities within the National Drug Abuse Treatment Clinical Trials Network | Paul M.RomanPh.D.abAmanda J.AbrahamPh.D.abTanja C.RothrauffPh.D.aHannah K.KnudsenPh.D.c |
| WoS | EVALUATION AND PROGRAM PLANNING | Other | 2010 | 10.1016/j.evalprogplan.2009.12.005 | Research collaboration in the discovery, development, and delivery networks of a statewide cancer coalition | Provan, Keith G.; Leischow, Scott J.; Keagy, Judith; Nodora, Jesse |
| WoS; PM | HEALTH EDUCATION & BEHAVIOR | Health | 2013 | 10.1177/1090198113492759 | Implications of Network Structure on Public Health Collaboratives | Retrum, Jessica H.; Chapman, Carrie L.; Varda, Danielle M. |
| WoS | BMJ OPEN | Health | 2019 | 10.1136/bmjopen-2019-028943 | Measuring coordination between women's self-help groups and local health systems in rural India: a social network analysis | Ruducha, J; Hariharan, D; Potter, J; Ahmad, D; Kumar, S; Mohanan, PS; Irani, L; Long, KNG |
| WoS; PM | EVALUATION AND PROGRAM PLANNING | Other | 2008 | 10.1016/j.evalprogplan.2008.06.002 | Collaboration and competition in a children's health initiative coalition: A network analysis | Valente, Thomas W.; Coronges, Kathryn A.; Stevens, Gregory D.; Cousineau, Michael R. |
| BuSCo | Public Performance & Management Review | Management | 2015 | 10.1080/15309576.2015.1031006 | Collaborative Performance as a Function of Network Members’ Perceptions of Success. | Varda, Danielle M.; Retrum, Jessica H. |
| WoS; PM | Journal of public health research | Health | 2012 | 10.4081/jphr.2012.e27 | An Exploratory Analysis of Network Characteristics and Quality of Interactions among Public Health Collaboratives. | Varda, Danielle M; Retrum, Jessica H |
| WoS; PM | JOURNAL OF COMMUNITY PSYCHOLOGY | Health | 2016 | 10.1002/jcop.21801 | NETWORK STRUCTURE, MULTIPLEXITY, AND EVOLUTION AS INFLUENCES ON COMMUNITY-BASED PARTICIPATORY INTERVENTIONS | Wang, Rong; Tanjasiri, Sora Park; Palmer, Paula; Valente, Thomas W. |
| PM | Preventing chronic disease | Health | 2015 | 10.5888/pcd12.150297 | Outcomes of Interorganizational Networks in Canada for Chronic Disease Prevention: Insights From a Concept Mapping Study, 2015 | Willis C, Kernoghan A, Riley B, Popp J, Best A, Milward HB. |
| WoS | CHILD ABUSE & NEGLECT | Health | 2009 | 10.1016/j.chiabu.2008.10.004 | Coordination between child welfare agencies and mental health service providers, children's service use, and outcomes | Bai, Yu; Wells, Rebecca; Hillemeier, Marianne M. |
| WoS; PM | HEALTH PROMOTION INTERNATIONAL | Health | 2010 | 10.1093/heapro/daq002 | Understanding the structure of community collaboration: the case of one Canadian health promotion network | Barnes, Martha; MacLean, Joanne; Cousens, Laura |
| WoS | NONPROFIT AND VOLUNTARY SECTOR QUARTERLY | Other | 2013 | 10.1177/0899764012451369 | Administrative Coordination in Nonprofit Human Service Delivery Networks: The Role of Competition and Trust | Bunger, Alicia C. |
| WoS | JOURNAL OF THE SOCIETY FOR SOCIAL WORK AND RESEARCH | Other | 2014 | 10.1086/679224 | Building Service Delivery Networks: Partnership Evolution Among Children's Behavioral Health Agencies in Response to New Funding | Bunger, Alicia C.; Doogan, Nathan J.; Cao, Yiwen |
| WoS | HEALTH CARE MANAGEMENT REVIEW | Health | 2014 | 10.1097/HMR.0b013e31828c8b76 | Coordinating nonprofit children's behavioral health services Clique composition and relationships | Bunger, Alicia C.; Gillespie, David F. |
| WoS | HUMAN SERVICE ORGANIZATIONS MANAGEMENT LEADERSHIP & GOVERNANCE | Administration | 2017 | 10.1080/23303131.2016.1184735 | Institutional and Market Pressures on Interorganizational Collaboration and Competition Among Private Human Service Organizations | Bunger, Alicia C.; McBeath, Bowen; Chuang, Emmeline; Collins-Camargo, Crystal |
| WoS; PM | PROGRESS IN COMMUNITY HEALTH PARTNERSHIPS-RESEARCH EDUCATION AND ACTION | Health | 2014 | 10.1353/cpr.2014.0001 | Utilization of an Interorganizational Network Analysis to Evaluate the Development of Community Capacity Among a Community-Academic Partnership | Clark, Heather R.; Ramirez, Albert; Drake, Kelly N.; Beaudoin, Christopher E.; Gainey, Whitney R.; Wendel, Monica L.; Outley, Corliss; Burdine, James N.; Player, Harold D. |
| WoS; PM | INTERNATIONAL JOURNAL OF INTEGRATED CARE | Health | 2014 | 10.5334/ijic.1138 | Network collaboration of organisations for homeless individuals in the Montreal region | Fleury, Marie-Josee; Grenier, Guy; Lesage, Alain; Ma, Nan; Ngui, Andre Ngamini |
| BuSCo; WoS; PM | Health Services Management Research | Health | 2019 | 10.1177/0951484818816417 | Evaluating communication as an essential precondition for inter-organisational learning in Governance Networks – The case of the Healthy Cities Network in Germany. | Grüb, Birgit; Martin, Sebastian |
| WoS | JOURNAL OF URBAN HEALTH-BULLETIN OF THE NEW YORK ACADEMY OF MEDICINE | Health | 2001 | 10.1093/jurban/78.3.468 | Interorganizational relationships among HIV/AIDS service organizations in Baltimore: A network analysis | Kwait, J; Valente, TW; Celentano, DD |
| PM | AUSTRALIAN JOURNAL OF PRIMARY HEALTH | Health | 2011 | 10.1071/PY10080 | Primary health care service delivery networks for the prevention and management of type 2 diabetes: using social network methods to describe interorganisational collaboration in a rural setting | McDonald J, Jayasuriya R, Harris MF. |
| WoS | PSYCHOSOCIAL INTERVENTION | Health | 2015 | 10.1016/j.psi.2015.07.005 | Strengthening suicide prevention networks: Interorganizational collaboration and tie strength | Menger, Lauren Marie; Stallones, Lorann; Cross, Jennifer Eileen; Henry, Kimberly Lynn; Chen, Peter Yu |
| WoS | VOLUNTAS | Other | 2014 | 10.1007/s11266-013-9403-4 | Humanitarian Interorganizational Information Exchange Network: How Do Clique Structures Impact Network Effectiveness? | Ngamassi, Louis; Maitland, Carleen; Tapia, Andrea H. |
| WoS; PM | HEALTH EDUCATION & BEHAVIOR | Health | 2003 | 10.1177/1090198103255366 | Building community capacity around chronic disease services through a collaborative interorganizational network | Provan, KG; Nakama, L; Veazie, MA; Teufel-Shone, NI; Huddleston, C |
| PM | Health Education and Behavior | Health | 2004 | 10.1177/1090198104264220 | Assessing interorganizational networks as a dimension of community capacity: illustrations from a community intervention to prevent lead poisoning | Singer, HH; Kegler MC |
| WoS; PM | DRUG AND ALCOHOL DEPENDENCE | Health | 2014 | 10.1016/j.drugalcdep.2014.01.006 | Reducing readmissions to detoxification: An interorganizational network perspective | Spear, Suzanne E. |
| WoS | SEXUALLY TRANSMITTED DISEASES | Other | 2007 | 10.1097/01.olq.0000223281.30734.b1 | An interagency network perspective on HIV prevention | Thomas, James C.; Isler, Malika R.; Carter, Craig; Torrone, Elizabeth |
| WoS | SOCIAL NETWORKS | Other | 2019 | 10.1016/j.socnet.2018.10.001 | Some days are better than others: Examining time-specific variation in the structuring of interorganizational relations | Amati, Viviana; Lomi, Alessandro; Mascia, Daniele |
| WoS | ORGANIZATIONAL RESEARCH METHODS | Management | 2021 | 10.1177/1094428119857469 | The Co-evolution of Organizational and Network Structure: The Role of Multilevel Mixing and Closure Mechanisms | Amati, Viviana; Lomi, Alessandro; Mascia, Daniele; Pallotti, Francesca |
| WoS | JOURNAL OF MANAGEMENT | Management | 2007 | 10.1177/0149206307305561 | Understanding collaboration outcomes from an extended resource-based view perspective: The roles of organizational characteristics, partner attributes, and network structures | Arya, Bindu; Lin, Zhiang (John) |
| WoS | JOURNAL OF HEALTH AND SOCIAL BEHAVIOR | Health | 1998 | 10.2307/2676345 | Organizational characteristics associated with agency position in community care networks | Banaszak-Holl, J; Allen, S; Mor, V; Schott, T |
| PM | Inquiry | Other | 2000 | PMID: 11111282 | The financial performance of hospitals belonging to health networks and systems | Bazzoli GJ, Chan B, Shortell SM, D'Aunno T. |
| WoS; PM | SOCIAL SCIENCE & MEDICINE | Health | 1998 | 10.1016/S0277-9536(97)10053-3 | Community-based trauma systems in the United States: An examination of structural development | Bazzoli, GJ; Harmata, R; Chan, CL |
| WoS; PM | JOURNAL OF INTERPROFESSIONAL CARE | Health | 2020 | 10.1080/13561820.2020.1712332 | Relational coordination in inter-organizational settings. How does lack of proximity affect coordination between hospital-based and community-based healthcare providers? | Bligaard Madsen S, Burau V. |
| PM | Statistical Medicine | Other | 2017 | 10.1002/sim.7301 | Bayesian exponential random graph modelling of interhospital patient referral networks | Caimo A, Pallotti F, Lomi A. |
| WoS | INTERNATIONAL JOURNAL OF INTEGRATED CARE | Health | 2016 | 10.5334/ijic.2462 | Does Telecare Improve Interorganisational Collaboration? | Christensen, Jannie Kristine Bang |
| BuSCo | Journal of Management Studies (Wiley-Blackwell) | Management | 2004 | 10.1111/j.1467-6486.2004.00424.x | Coordination Networks Within and Across Organizations: A Multi-level Framework. | Gittell, Jody Hoffer; Weiss, Leigh |
| WoS; PM | CANCER EPIDEMIOLOGY BIOMARKERS & PREVENTION | Health | 1994 | PMID: 8019377 | USING A COMMUNITY CANCER-TREATMENT TRIALS NETWORK FOR CANCER PREVENTION AND CONTROL RESEARCH - CHALLENGES AND OPPORTUNITIES | KALUZNY, AD; LACEY, LM; WARNECKE, R; MORRISSEY, JP; SONDIK, E; FORD, L |
| PM | BMC HEALTH SERVICES RESEARCH | Health | 2017 | 10.1186/s12913-017-2096-4 | Coordination between primary and secondary care: the role of electronic messages and economic incentives | La Rocca A, Hoholm T. |
| BuSCo | Human Relations | Other | 1989 | 10.1177/001872678904201204 | Interorganizational Systems in Public Service Delivery: A New Application of the Dynamic Network Framework. | Lawless, Michael W.; Moore, Rita A. |
| WoS | SOCIAL NETWORKS | Other | 2012 | 10.1016/j.socnet.2010.10.005 | Relational collaboration among spatial multipoint competitors | Lomi, A.; Pallotti, F. |
| WoS; PM | BMC HEALTH SERVICES RESEARCH | Health | 2018 | 10.1186/s12913-018-3474-2 | Exploring role clarity in interorganizational spread and scale-up initiatives: the 'INSPIRED' COPD collaborative | Ly, Olivia; Sibbald, Shannon L.; Verma, Jennifer Y.; Rocker, Graeme M. |
| BuSCo | International Journal of Public Administration | Administration | 2014 | 10.1080/01900692.2014.944993 | Exploring the Effect of Network Governance Models on Health-Care Systems Performance. | Marafioti, Elisabetta; Mariani, Laura; Martini, Mattia |
| PM | HEALTH CARE MANAGEMENT REVIEW | Health | 2013 | 10.1097/HMR.0b013e31824ccab8 | Dynamics of hospital competition: social network analysis in the Italian National Health Service | Mascia, D.; Di Vincenzo, F. |
| WoS; PM | SOCIAL SCIENCE & MEDICINE | Health | 2015 | 10.1016/j.socscimed.2015.03.029 | Effect of hospital referral networks on patient readmissions | Mascia, Daniele; Angeli, Federica; Di Vincenzo, Fausto |
| WoS | HEALTH POLICY | Health | 2012 | 10.1016/j.healthpol.2012.02.011 | Dynamic analysis of interhospital collaboration and competition: Empirical evidence from an Italian regional health system | Mascia, Daniele; Di Vincenzo, Fausto; Cicchetti, Americo |
| WoS | REGIONAL STUDIES | Other | 2017 | 10.1080/00343404.2016.1185517 | Don't stand so close to me: competitive pressures, proximity and inter-organizational collaboration | Mascia, Daniele; Pallotti, Francesca; Angeli, Federica |
| BuSCo; WoS | British Journal of Management | Management | 2009 | 10.1111/j.1467-8551.2008.00565.x | The Coexistence of Competition and Cooperation between Networks: Implications from Two Taiwanese Healthcare Networks. | Peng, Tzu‐Ju Ann; Bourne, Mike |
| PM | HEALTH CARE MANAGEMENT REVIEW | Health | 2006 | 10.1097/00004010-200610000-00007 | Benefiting from networks by occupying central positions: an empirical study of the Taiwan health care industry | Peng, Tzu‐Ju Ann; Lo, Fang-Yi; Lin, Chin-Sien; Yu, Chwo-Ming Joseph |
| WoS | ADMINISTRATIVE SCIENCE QUARTERLY | Administration | 1995 | 10.2307/2393698 | A PRELIMINARY THEORY OF INTERORGANIZATIONAL NETWORK EFFECTIVENESS - A COMPARATIVE-STUDY OF 4 COMMUNITY MENTAL-HEALTH SYSTEMS | PROVAN, KG; MILWARD, HB |
| WoS | International Journal of Managing Projects in Business | Management | 2019 | 10.1108/IJMPB-10-2018-0230 | Asymmetry of stakeholders' perceptions as an obstacle for collaboration in inter-organizational projects The case of medicine traceability projects | Romero-Torres, A |
| BuSCo; WoS | Public Management Review | Management | 2010 | 10.1080/14719037.2010.488860 | Public-Private Partnerships in Health Services Delivery. | Singh, Avantika; Prakash, Gyan |
| WoS | JOURNAL OF RURAL HEALTH | Health | 1997 | 10.1111/j.1748-0361.1997.tb00846.x | Delivering care to rural HIV/AIDS patients | Topping, S; Hartwig, LC |
| WoS | SOCIAL NETWORKS | Other | 2016 | 10.1016/j.socnet.2015.06.005 | The embeddedness of organizational performance: Multiple Membership Multiple Classification Models for the analysis of multilevel networks | Tranmer, Mark; Pallotti, Francesca; Lomi, Alessandro |
| WoS | HEALTH CARE MANAGEMENT REVIEW | Health | 2010 | 10.1097/HMR.0b013e3181a93f8d | Better to receive than to give? Interorganizational service arrangements and hospital performance | Trinh, Hanh Q.; Begun, James W.; Luke, Roice D. |
| BuSCo; WoS; PM | Health Services Management Research | Health | 2021 | 10.1177/0951484820971456 | Inter-organizational collaboration between healthcare providers | van der Schors, Wouter; Roos, Anne-Fleur; Kemp, Ron; Varkevisser, Marco |
| WoS | BMC HEALTH SERVICES RESEARCH | Health | 2013 | 10.1186/1472-6963-13-296 | Care pathways across the primary-hospital care continuum: using the multi-level framework in explaining care coordination | Van Houdt, Sabine; Heyrman, Jan; Vanhaecht, Kris; Sermeus, Walter; De Lepeleire, Jan |
| WoS | MEDICAL CARE RESEARCH AND REVIEW | Health | 2007 | 10.1177/1077558707301166 | Adapting a dynamic model of interorganizational cooperation to the health care sector | Wells, Rebecca; Weiner, Bryan J. |
| WoS | SOCIAL SCIENCE & MEDICINE | Health | 2017 | 10.1016/j.socscimed.2017.05.051 | Coopetition in health care: A multi-level analysis of its individual and organizational determinants | Westra, Daan; Angeli, Federica; Carree, Martin; Ruwaard, Dirk |
| WoS | SOCIAL SCIENCE & MEDICINE | Health | 2016 | 10.1016/j.socscimed.2016.06.019 | Understanding specialist sharing: A mixed-method exploration in an increasingly price-competitive hospital market | Westra, Daan; Angeli, Federica; Jatautaite, Evelina; Carree, Martin; Ruwaard, Dirk |
| WoS | JOURNAL OF PUBLIC ADMINISTRATION RESEARCH AND THEORY | Administration | 2017 | 10.1093/jopart/muw061 | Voices from the Frontline: Network Participation and Local Support for National Policy Reforms | Zhu, Ling |
| WoS; PM | DISASTER MEDICINE AND PUBLIC HEALTH PREPAREDNESS | Health | 2017 | 10.1017/dmp.2016.142 | Informal Networks in Disaster Medicine | Bdeir, Fadl; Crawford, John W.; Hossain, Liaquat |
| WoS | SAFETY SCIENCE | Other | 2016 | 10.1016/j.ssci.2016.01.006 | From linearity to complexity: Emergent characteristics of the 2006 Avian Influenza Response System in Turkey | Celik, Suleyman; Corbacioglu, Sitki |
| WoS | HEALTH & SOCIAL CARE IN THE COMMUNITY | Health | 2001 | 10.1046/j.1365-2524.2001.00305.x | Negotiating and managing partnership in primary care | Charlesworth, J |
| WoS | HEALTH PROMOTION INTERNATIONAL | Health | 1997 | 10.1093/heapro/12.1.9 | Joint working and the production of a City Health Plan: The Liverpool experience | Costongs, C; Springett, J |
| WoS | SOCIAL SERVICE REVIEW | Other | 1992 | 10.1086/603947 | ORGANIZING AIDS SERVICE CONSORTIA - LEAD AGENCY IDENTITY AND CONSORTIUM COHESION | FLEISHMAN, JA; MOR, V; PIETTE, JD; ALLEN, SM |
| WoS | DRUG AND ALCOHOL DEPENDENCE | Health | 2009 | 10.1016/j.drugalcdep.2009.01.001 | Measuring collaboration and integration activities in criminal justice and substance abuse treatment agencies | Fletcher, Bennett W.; Lehman, Wayne E. K.; Wexler, Harry K.; Melnick, Gerald; Taxman, Faye S.; Young, Douglas W. |
| WoS | AMERICAN JOURNAL OF COMMUNITY PSYCHOLOGY | Health | 2001 | 10.1023/A:1012915631956 | Facilitating interorganizational collaboration: The contributions of interorganizational alliances | Foster-Fishman, PG; Salem, DA; Allen, NA; Fahrbach, K |
| WoS | JOURNAL OF COMMUNITY PSYCHOLOGY | Health | 1996 | 10.1002/(SICI)1520-6629(199607)24:3<275::AID-JCOP7>3.0.CO;2-W | Structure and change in child mental health service delivery networks | Johnsen, MC; Morrissey, JP; Calloway, MO |
| BuSCo | Public Administration | Administration | 2006 | 10.1111/j.0033-3298.2006.00500.x | Public-Nonprofit Partnerships for Collective Action in Dynamic Contexts of Emergencies. | Kapucu, Naim |
| WoS | KNOWLEDGE MANAGEMENT RESEARCH & PRACTICE | Other | 2015 | 10.1057/kmrp.2014.3 | A managerial view of the knowledge flows of a health-care system | Laihonen, Harri |
| WoS | DRUG AND ALCOHOL DEPENDENCE | Health | 2009 | 10.1016/j.drugalcdep.2009.01.004 | Organizational factors and collaboration and integration activities in criminal justice and drug abuse treatment agencies | Lehman, Wayne E. K.; Fletcher, Bennett W.; Wexler, Harry K.; Melnick, Gerald |
| WoS | BMC PUBLIC HEALTH | Health | 2008 | 10.1186/1471-2458-8-382 | Diagnosis of sustainable collaboration in health promotion - a case study | Leurs, Mariken T. W.; Mur-Veeman, Ingrid M.; van der Sar, Rosalie; Schaalma, Herman P.; de Vries, Nanne K. |
| PM | HEALTH SERVICES RESEARCH | Health | 1993 | PMID: 8407338 | Interorganizational exchanges as performance markers in a community cancer network. | McKinney MM, Morrissey JP, Kaluzny AD. |
| BuSCo; WoS | Administration in Social Work | Administration | 1998 | 10.1300/J147v22n04_01 | Motivation and Reward in Nonprofit Interorganizational Collaboration in Low-Income Neighborhoods. | Mulroy, Elizabeth A.; Shay, Sharon |
| PM | American Journal of Public Health | Health | 1986 | 10.2105/ajph.76.7.755 | Referral patterns to and from inpatient psychiatric services: a social network approach | Nakao K, Milazzo-Sayre LJ, Rosenstein MJ, Manderscheid RW. |
| BuSCo | Journal of Behavioral Health Services & Research | Health | 2016 | 10.1007/s11414-014-9409-8 | How Community Organizations Promote Continuity of Care for Young People with Mental Health Problems. | Polgar, Michael; Cabassa, Leopoldo; Morrissey, Joseph |
| BuSCo; WoS | Administration & Society | Administration | 2007 | 10.1177/0095399706297212 | Governance, Power, and Mandated Collaboration in an Interorganizational Network. | Rodríguez, Charo; Langley, Ann; Denis, Jean-Louis; Béland, François |
| BuSCo | Human Service Organizations: Management, Leadership & Governance | Administration | 2014 | 10.1080/03643107.2013.828005 | Networking for Policy Advocacy: Identifying Predictors of Advocacy Success Among Human Service Organizations. | Ruggiano, Nicole; Taliaferro, JocelynDeVance; Dillon, FrankR.; Granger, Ted; Scher, Jessica |
| WoS | GOVERNMENT INFORMATION QUARTERLY | Other | 2007 | 10.1016/j.giq.2007.04.001 | Towards end-to-end government performance management: Case study of interorganizational information integration in emergency medical services (EMS) | Schooley, Ben L.; Horan, Thomas A. |
| WoS | JMIR medical informatics | Health | 2013 | 10.2196/medinform.2510 | Bridging organizational divides in health care: an ecological view of health information exchange. | Unertl, Kim M; Johnson, Kevin B; Gadd, Cynthia S; Lorenzi, Nancy M |
| WoS | JOURNAL OF PRIMARY PREVENTION | Health | 2010 | 10.1007/s10935-010-0203-y | Interorganizational Network Changes Among Health Organizations in the Brazos Valley, Texas | Wendel, Monica L.; Prochaska, John D.; Clark, Heather R.; Sackett, Shawta; Perkins, Keith |
| BuSCo | American Journal of Public Health | Health | 1993 | 10.2105/AJPH.83.4.561 | Activating Communities for Health Promotion: A Process Evaluation Method. | Wickizer, Thomas M.; Von Korff, Michael; Cheadle, Allen; Maeser, Jennifer; Wagner, Edward H.; Pearson, David; Beery, William; Psaty, Bruce M. |
| WoS; PM | Healthcare policy = Politiques de sante | Health | 2016 | PMID: 27585030 | Mapping Collaborative Relations among Canada's Chronic Disease Prevention Organizations. | Contandriopoulos, Damien; Hanusaik, Nancy; Maximova, Katerina; Paradis, Gilles; O'Loughlin, Jennifer L |
| WoS; PM | HEALTH RESEARCH POLICY AND SYSTEMS | Health | 2021 | 10.1186/s12961-021-00792-0 | A systems approach to the exploration of research activity and relationships within a local authority | Fynn, Judith F.; Jones, John; Jones, Andy |
| PM | INTERNATIONAL JOURNAL OF INTEGRATED CARE | Health | 2021 | 10.5334/ijic.5635 | Balancing Pragmatism and Sustainability: A Case Study of an Interorganisational Network to Improve Integrated Care for the Elderly | Hallberg, A., Winblad, U. & Fredriksson, M. |
| PM | PLoS Currents | Other | 2012 | 10.1371/4f7f57285b804 | Local Public Health System Response to the Tsunami Threat in Coastal California following the TÅhoku Earthquake | Hunter JC, Crawley AW, Petrie M, Yang JE, AragÃ³n TJ. |
| BuSCo | Academy of Management Proceedings & Membership Directory | Management | 2002 | 10.5465/APBPP.2002.7519436 | THE EVOLUTION OF INTERORGANIZATIONAL NETWORK RELATIONSHIPS OVER TIME. | ISETT, KIMBERLEY ROUSSIN;PROVAN, KEITH G. |
| WoS; PM | Preventing chronic disease | Health | 2004 | PMID: 15670440 | Interorganizational relationships within state tobacco control networks: a social network analysis. | Krauss, Melissa; Mueller, Nancy; Luke, Douglas |
| WoS; PM | International journal of integrated care | Health | 2007 | 10.5334/ijic.199 | Clustering and inertia: structural integration of home care in Swedish elderly care. | Olof Hedman, Nils; Johansson, Roine; Rosenqvist, Urban |
| BuSCo | International Journal of Networking & Virtual Organisations | Other | 2009 | 10.1504/IJNVO.2009.023807 | The policy, structure and evaluation of long-term care networks: the differentiated requirements referred to the example of integrated long-term care in Austria. | Ostermann, Herwig; Staudinger, Bettina; Staudinger, Oskar; Kern, Katharina; Them, Christa; Staudinger, Roland |
| BuSCo | Global Health Governance | Health | 2018 | n/a | Transnational Human Rights Organizing and Global Health Governance, 1963-2013. | Plummer, Samantha; Smith, Jackie; Hughes, Melanie |
| BuSCo | Academy of Management Annual Meeting Proceedings | Management | 2009 | 10.5465/AMBPP.2009.44243406 | RESOURCE TANGIBILITY AND THE EVOLUTION OF PUBLIC NETWORK SUB-STRUCTURES. | PROVAN, KEITH G.; HUANG, KUN |
| BuSCo; WoS; PM | Journal of health and human services administration | Health | 2002 | PMID: 15189000 | Interorganizational networks: using a theoretical model to predict effectiveness of rural health care delivery networks. | Schumaker, Alice M |
| WoS | Services Industries Journal | Other | 2013 | 10.1080/02642069.2013.815729 | Performance impacts of interorganizational cooperation: a transaction cost perspective | Sui-Ha Yu, Ming-Yu Chen |
| BuSCo | Economics & Management / Ekonomia i Zarzadzanie | Management | 2017 | 10.1515/emj-2017-0011 | Network Analysis Approach to Stroke Care and Assistance Provision: An Empirical Study. | Szczygiel, Nina; Santana, Silvina |
| PM | Journal of Biomedical Informatics | Other | 2007 | 10.1016/j.jbi.2006.11.001 | Information infrastructure for inter-organizational mental health services: an actor network theory analysis of psychiatric rehabilitation | Timpka, T;Bang, M;Delbanco, T; Walker, J |
